# Dabigatran Attenuates Osteoporosis by Balancing Osteoblastogenesis and Osteoclastogenesis by Targeting PRKAB1 and RELA

**DOI:** 10.34133/research.1423

**Published:** 2026-09-04

**Authors:** Feiyu Chen, Yifeng Shang, Kaiwen Liu, Qianli Yin, Yong Hou, Menglin Cong, Jie Zhao, Baoliang Zhang, Lisha Sun, Meng Si, Hecheng Ma

**Affiliations:** ^1^ Department of Orthopedics, Qilu Hospital of Shandong University, Jinan 250012, China.; ^2^ Department of Ophthalmology, Shandong Provincial Hospital Affiliated to Shandong First Medical University, Jinan 250021, China.; ^3^ Department of Oncology, Shengjing Hospital of China Medical University, Shenyang 110000, China.

## Abstract

Imbalances between osteoblastogenesis and osteoclastogenesis represent the fundamental pathological feature of primary osteoporosis; however, safe and cost-effective therapies that simultaneously promote bone formation and suppress bone resorption are lacking. Given the central roles of runt-related transcription factor 2 and the nuclear factor κB pathway in bone remodeling, we performed a phenotypic screen of a Food and Drug Administration-approved drug library to identify dual-acting regulators of bone homeostasis and identified the orally approved anticoagulant dabigatran (DAB) as a novel candidate. In vivo, DAB administration markedly attenuated bone loss and preserved bone microarchitecture in both ovariectomized and aged mouse models of osteoporosis. In vitro, DAB not only inhibited receptor activator of nuclear factor κB ligand-induced osteoclastogenesis from bone-marrow-derived macrophages but also directly enhanced the osteogenic differentiation of bone marrow mesenchymal stem cells. Mechanistically, using limited proteolysis-coupled mass spectrometry, we identified protein kinase adenosine-monophosphate-activated noncatalytic subunit β1 and v-Rel reticuloendotheliosis viral oncogene homolog A as direct targets of DAB. In bone marrow mesenchymal stem cells, DAB binds to protein kinase adenosine-monophosphate-activated noncatalytic subunit β1 via the alanine-77 residue, leading to adenosine-monophosphate-activated protein kinase activation, subsequent mechanistic target of rapamycin complex 1 inhibition, enhanced autophagic flux, and ultimately promoted osteogenesis; in osteoclast precursors, DAB interacts with v-Rel reticuloendotheliosis viral oncogene homolog A at arginine-50, resulting in suppression of the nuclear factor κB pathway and attenuated osteoclast differentiation. Collectively, our findings reposition DAB as a promising therapeutic candidate for restoring osteoblast–osteoclast balance and maintaining skeletal homeostasis while establishing a novel single-molecule, dual-target pharmacological strategy and providing novel mechanistic insights for bone metabolic disease treatment.

## Introduction

As the global population ages, maintaining skeletal integrity against osteoporotic decline has become a defining challenge for longevity and quality of life [1]. Osteoporosis, a prevalent age-related metabolic bone disease, is characterized by reduced bone mass and deteriorated bone microarchitecture, leading to increased fracture risk and imposing a substantial global health burden [2,3]. Bone homeostasis is maintained by a precise balance between osteoblast-mediated bone formation and osteoclast-mediated bone resorption [4–6]. Osteoblastogenesis refers to the stepwise differentiation of bone marrow mesenchymal stem cells (BMSCs) into matrix-secreting, mineralizing mature osteoblasts, a process orchestrated by core transcriptional regulators such as runt-related transcription factor 2 (RUNX2) and osterix (OSX) [7]. In parallel, osteoclastogenesis involves the fusion and maturation of myeloid-derived mononuclear precursors into multinucleated, bone-resorbing osteoclasts, which is tightly driven by receptor activator of nuclear factor κB (NF-κB) ligand (RANKL) and its downstream NF-κB signaling cascades [8]. Disruption of this tightly coupled differentiation program, characterized by suppressed bone formation or excessive bone resorption, directly drives pathological bone loss [9]. In primary osteoporosis, such as the postmenopausal and senile forms, this balance is disrupted [10].

Currently, most available antiosteoporosis drugs exhibit single-action mechanisms: Antiresorptive agents (e.g., bisphosphonates and denosumab) primarily suppress osteoclast activity, whereas anabolic drugs (e.g., teriparatide and romosozumab) mainly promote osteoblast formation [11]. However, long-term use of these agents may be associated with adverse effects, such as osteonecrosis of the jaw with bisphosphonates or potential cardiovascular risks with romosozumab [12,13]. Furthermore, many are costly and require parenteral administration, which collectively hinder accessibility and adherence [14,15]. Furthermore, the gut–bone axis has emerged as another attractive therapeutic strategy for osteoporosis, offering an orally available, multipathway approach that concurrently modulates the immune, endocrine, and metabolic regulation of the bone; however, clinical translation of this strategy remains constrained by marked interindividual heterogeneity of the gut microbiome, poorly defined effector strains, and a scarcity of high-level clinical evidence [16,17]. Thus, the field has converged on a pivotal therapeutic goal: the development of a single, orally available agent that can fundamentally reboot bone homeostasis by concurrently amplifying anabolic and suppressing catabolic pathways.

To directly address this therapeutic goal and circumvent the decade-long timelines of traditional drug development, we turned to drug repurposing, a strategy accelerated by modern phenotypic screening or computational biology, which leverages existing clinical safety data to rapidly deliver affordable, orally administered candidates [18]. Small-molecule drugs, owing to their advantages such as oral convenience, relatively low cost, and well-defined mechanisms of action, are particularly suitable for the long-term management of chronic conditions such as osteoporosis [19]. Therefore, we performed a phenotypic screen of a Food and Drug Administration (FDA)-approved drug library, targeting the master regulators of bone formation (RUNX2) and resorption (NF-κB), to identify compounds with dual activity to restore bone homeostasis. In this screening, the oral anticoagulant dabigatran (DAB) was identified as a previously unrecognized dual-action compound that promotes bone formation and inhibits bone resorption.

DAB is a direct thrombin inhibitor widely used for the prevention and treatment of thrombotic diseases. As the first approved oral direct thrombin inhibitor, DAB offers predictable pharmacokinetics, fewer dietary and drug interactions, and no requirement for routine coagulation monitoring compared with classic vitamin K antagonists, and it is broadly prescribed for stroke prevention in atrial fibrillation and the treatment of venous thromboembolism [20]. Accumulating clinical evidence suggests potential associations between DAB use and favorable skeletal outcomes; however, preclinical evidence regarding its direct impact on osteoblast and osteoclast differentiation remains scarce [21]. Notably, conventional anticoagulants such as unfractionated heparin and warfarin have long been associated with adverse skeletal effects and increased fracture risk [22]. In contrast, whether DAB exerts distinct regulatory effects on bone metabolism and the underlying molecular mechanisms remains poorly characterized. In this study, we systematically validated that DAB exerts profound protective effects against osteoporosis in vivo and promotes a pro-osteogenic, antiosteoclastic cellular phenotype in vitro. Using limited proteolysis-coupled mass spectrometry (LiP-MS), we identified adenosine monophosphate (AMP)-activated protein kinase (AMPK) subunit protein kinase AMP-activated noncatalytic subunit β1 (PRKAB1) and v-Rel reticuloendotheliosis viral oncogene homolog A (RELA) as direct molecular targets of DAB. We further elucidated that in BMSCs, DAB binds to PRKAB1 at Ala77, activating the AMPK/mechanistic target of rapamycin complex 1 (mTORC1) pathway, and stimulates autophagic flux to drive osteogenic differentiation. Conversely, in osteoclast precursors, DAB interacts with RELA at Arg50, suppressing the NF-κB signaling pathway and inhibiting osteoclastogenesis. Collectively, the results of this work transcend drug repurposing by establishing DAB as a pharmacological prototype and a chemical probe for bone anabolism. Our findings validate the feasibility of a “1-molecule, 2-target, 2-cell-type” strategy for systemic skeletal rejuvenation, thereby establishing a novel and targetable paradigm for systemically reprogramming bone homeostasis.

## Results

### Identification of DAB as a potential therapeutic agent for osteoporosis

To identify agents with dual bioactivities (osteogenic promotion and osteoclastogenic inhibition), we executed a tiered 3-round screening process using a library of 201 FDA-approved clinical medications [23,24]. For the initial osteogenesis-focused screen, the RUNX2 transactivation firefly luciferase (pRUNX2-TA-Luc) reporter plasmid, a well-established surrogate for osteogenic differentiation, was utilized to prioritize 20 lead compounds from the 201-member library (Fig. S1A). Building on this subset, the second round of screening targeted osteoclastogenesis inhibition via the pNF-κB-Luc reporter plasmid, a canonical marker of osteoclast activation; this step further refined the candidate pool to 4 agents: DAB, fexofenadine, delanzumab, and bezitramide (Fig. S1B). Functional validation was performed in the third round, where alkaline phosphatase (ALP) activity, a critical functional readout of osteogenic potential, was assessed in immortalized mouse BMSCs. Through this sequential, function-driven screening pipeline, DAB was ultimately identified as the lead compound with robust dual activity (Fig. S1C). To assess its safety, we evaluated the cytotoxicity of DAB on BMSCs using a Cell Counting Kit-8 (CCK-8) assay across a range of concentrations (0, 1, and 10 μM) and time points (24, 48, and 72 h). DAB exhibited no significant cytotoxicity within this concentration and exposure range (Fig. S1D). To evaluate the in vivo safety of DAB, mice received daily oral gavage of DAB at various concentrations for 60 d, while control animals were administered equivalent volumes of vehicle (Ham’s F-12 nutrient mixture [F-12] containing dimethyl sulfoxide [DMSO]). Sixty days later, mice were euthanized, and major organs were collected for histopathological analysis by hematoxylin and eosin (H&E) staining. No pathological lesions or tissue damage were detected in any of the groups. Histological assessment confirmed normal organ architecture: Cardiac muscle fibers retained cross-striations and centrally located round nuclei; hepatocytes were polygonal with distinct lobular organization and blood-filled sinusoids; pulmonary alveolar structures remained intact, accompanied by unremarkable bronchial epithelium and vascular walls; and renal tissue exhibited healthy glomeruli and tubules without evidence of injury (Fig. S1E). To further characterize systemic toxicity, we conducted serum biochemical analyses after the 60-d treatment course. All major biochemical parameters including hepatic function markers (alanine transaminase and aspartate transaminase), renal function markers (creatinine and blood urea nitrogen), and metabolic indicators (total cholesterol, triglyceride, and glucose) remained within normal physiological limits across all groups (Fig. S1F). These quantitative data, in agreement with the histopathological findings, collectively demonstrate that DAB, at the therapeutic concentrations used in this study, does not cause detectable hepatotoxicity, nephrotoxicity, or systemic metabolic disturbances.

### DAB promotes bone formation and attenuates accelerated bone loss in vivo

**Fig. 1.**
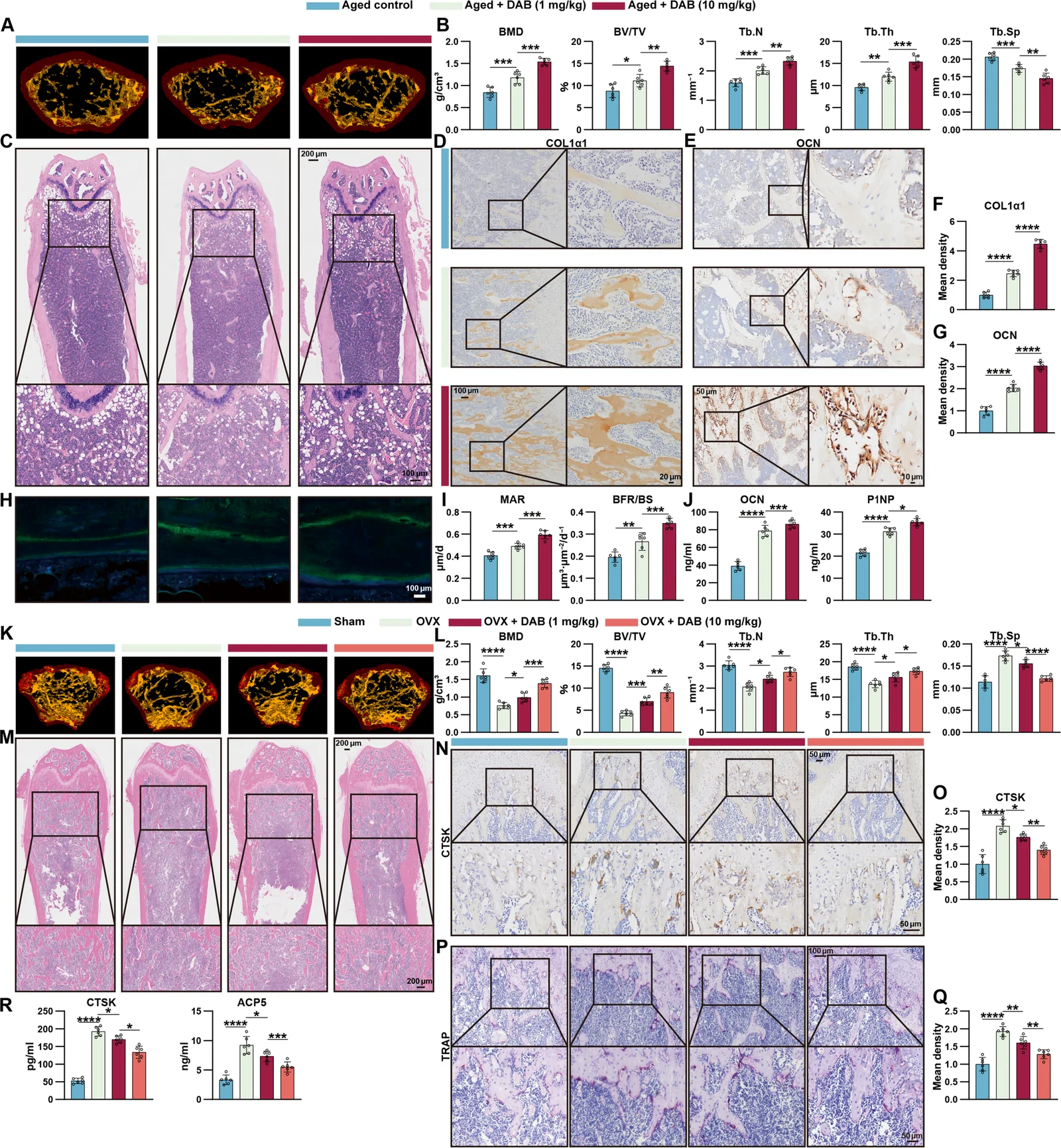
Dabigatran (DAB) promotes bone formation and attenuates accelerated bone loss in vivo. (A) Representative micro-computed-tomography (CT) reconstruction images of distal femurs obtained from aged male mice under 3 experimental groups: aged control, aged + DAB (1 mg/kg), and aged + DAB (10 mg/kg). (B) Quantitative analysis of bone microarchitecture parameters in aged mice, including bone mineral density (BMD), bone volume/tissue volume (BV/TV), trabecular number (Tb.N), trabecular thickness (Tb.Th), and trabecular separation (Tb.Sp). (C) Hematoxylin and eosin (H&E) staining of distal femur sections from aged mice. Scale bars, 200 and 100 μm. (D to G) Immunohistochemistry (IHC) staining of collagen type Iα1 (COL1α1) and osteocalcin (OCN) in distal femur sections from aged mice (scale bars, 100, 20, 50, and 10 μm) and mean optical density quantification. (H and I) Calcein double-labeling fluorescence staining to assess bone formation in aged mice (scale bar, 100 μm) and histomorphometric analysis of MAR and BFR/BS. (J) Serum levels of bone formation markers OCN and procollagen I N-terminal propeptide (P1NP) in aged mice. (K) Representative micro-CT reconstruction images of distal femurs from ovariectomized (OVX) female mice with sham, OVX, OVX + DAB (1 mg/kg), and OVX + DAB (10 mg/kg) groups. (L) Quantitative analysis of bone microarchitecture parameters in OVX mice, including BMD, BV/TV, Tb.N, Tb.Th, and Tb.Sp. (M) H&E staining of distal femur sections from OVX mice. Scale bars, 200 μm. (N and O) IHC staining of cathepsin K (CTSK) in distal femur sections from OVX mice (scale bars, 50 μm) and mean optical density quantification. (P and Q) Tartrate-resistant acid phosphatase (TRAP) staining of distal femur sections from OVX mice (scale bars, 100 and 50 μm) and quantification of the mean density of TRAP-positive staining in OVX mice. (R) Serum levels of bone resorption markers CTSK and acid phosphatase 5 (ACP5) in OVX mice. Data are presented as means ± SD (*n* = 6 mice per group). One-way ANOVA was used. Statistical significance: *P < 0.05, **P < 0.01, ***P < 0.001, and ****P < 0.0001; ns, not significant.

To explore the ability of DAB to alleviate age-related osteoporosis, we administered DAB (at doses of 1 or 10 mg/kg) to 24-month-old naturally senescent mice via daily oral gavage for 60 consecutive days. Compared with the control group, posttreatment micro-computed-tomography (CT) analysis of the distal femur showed that DAB increased quantitative bone mass and improved microarchitectural structure (Fig. 1A), and quantitative assessments revealed that DAB significantly increased bone mineral density (BMD), bone volume/tissue volume (BV/TV), trabecular thickness (Tb.Th), and trabecular number (Tb.N) and reduced trabecular separation (Tb.Sp) (Fig. 1B). Histological examination via H&E staining aligned with these results, revealing a larger trabecular area in DAB-treated mice (Fig. 1C). The osteogenic effect of DAB was further confirmed by immunohistochemistry (IHC) staining, which revealed notable increases in osteocalcin (OCN)-positive osteoblasts and collagen type Iα1 (COL1α1) expression in the DAB groups (Fig. 1D to G). To assess dynamic bone formation, we performed calcein double labeling, and fluorescence microscopy with subsequent histomorphometric analysis verified that DAB treatment significantly increased the mineral apposition rate (MAR) and bone formation rate per bone surface (BFR/BS) (Fig. 1H and I). Finally, in support of its bone-building properties, serum enzyme-linked immunosorbent assay (ELISA) demonstrated that DAB substantially increased the levels of osteogenic markers, including procollagen I N-terminal propeptide (P1NP) and OCN (Fig. 1J). Collectively, these data confirm that DAB promotes bone formation and improves bone mass and quality in aged mice.

To assess the in vivo bone-protective activity of DAB, we used an ovariectomized (OVX) mouse model of postmenopausal osteoporosis: 12-week-old female mice underwent OVX surgery, followed by a 60-d period to allow progressive bone loss, and were then given daily oral gavage of either vehicle control or DAB (at doses of 1 and 10 mg/kg) for an additional 60 d. Micro-CT assessment of the distal femur revealed that DAB treatment, particularly at the higher dose of 10 mg/kg, markedly mitigated OVX-induced bone loss (Fig. 1K), and quantitative analyses further revealed that DAB administration led to substantial changes in the BMD, BV/TV, Tb.Th, Tb.N, and Tb.Sp compared with those in the OVX control group (Fig. 1L). Histological examination via H&E staining aligned with these observations, further confirming the protective effect of DAB against OVX-accelerated bone loss (Fig. 1M). IHC staining revealed that DAB reduced cathepsin K (CTSK) expression in OVX mice (Fig. 1N and O), and tartrate-resistant acid phosphatase (TRAP) staining also revealed a significant decrease in the number of osteoclasts on trabecular bone surfaces in DAB-treated mice (Fig. 1P and Q). Finally, serum ELISA revealed notably lower levels of bone-related biomarkers, including CTSK and acid phosphatase 5 (ACP5), in DAB-treated mice than in OVX mice (Fig. 1R). Collectively, these data indicate that DAB treatment effectively enhances bone formation and inhibits bone resorption, thereby preventing OVX-induced bone loss in mice.

### DAB suppresses RANKL-induced osteoclastogenesis and regulates osteoblastogenesis

**Fig. 2.**
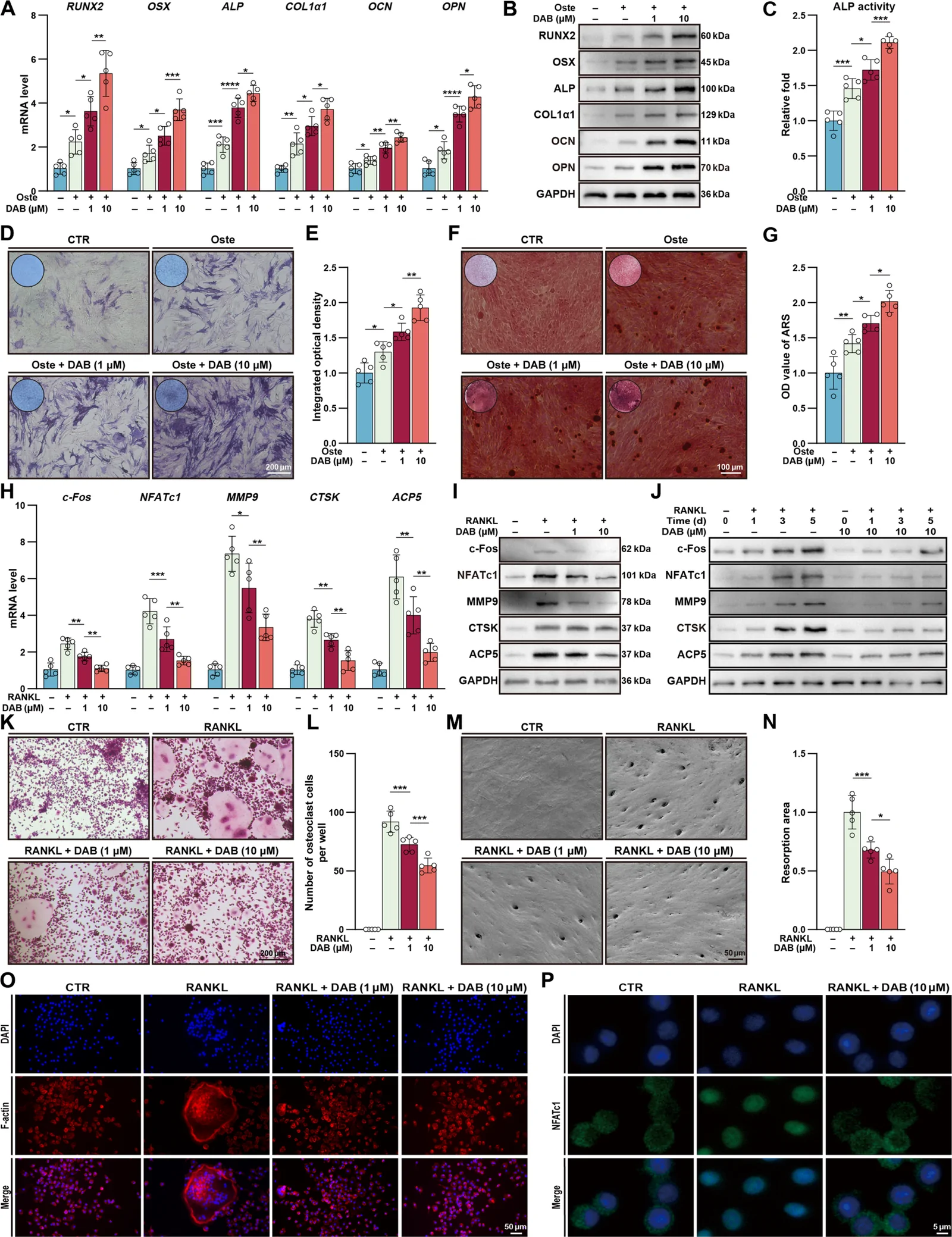
Dabigatran (DAB) suppresses receptor activator of nuclear factor κB ligand (RANKL)-induced osteoclastogenesis and regulates osteoblastogenesis. (A) Quantitative real-time reverse transcription polymerase chain reaction (qRT-PCR) analysis of osteogenic markers (*RUNX2*, *OSX*, *ALP*, *COL1α1*, *OCN*, and *OPN*) mRNA expression in bone marrow mesenchymal stem cells (BMSCs) after osteogenic induction with DAB for 3 d. (B) Western blot analysis of osteogenic marker proteins in BMSCs treated with DAB for 5 d. (C) Alkaline phosphatase (ALP) activity assay in BMSCs after 7 d of osteogenic induction. (D and E) ALP staining (D) (scale bar, 200 μm) and quantitative analysis of ALP-positive area (E). (F and G) ARS staining (F) (scale bar, 100 μm) and quantification of optical density (OD) value (G). (H and I) Bone-marrow-derived macrophages (BMDMs) were treated with the indicated concentrations of DAB and stimulated with RANKL for the specified durations. (H) mRNA levels of osteoclastogenic markers (*c-Fos*, *NFATc1*, *MMP9*, *ACP5*, and *CTSK*) were determined by qRT-PCR. (I) Protein expression of the indicated markers was analyzed by Western blotting. (J) BMDMs were treated with DAB and RANKL for different time points, and protein expression was analyzed by Western blotting. (K and L) BMDMs were treated with DAB and RANKL, followed by tartrate-resistant acid phosphatase (TRAP) staining (scale bar, 200 μm) (K); the number and area of TRAP-positive multinucleated cells (nuclei ≥ 3) were quantified (L). (M and N) Bone resorption assays performed on bovine femoral bone slices further confirmed the suppressive effect of DAB on osteoclastic bone resorption activity (scale bar, 50 μm) (M); the resorption area was quantified (N). (O) Cytoskeletal structure was visualized by rhodamine–phalloidin staining of F-actin rings (red). Nuclei were stained with 4′,6-diamidino-2-phenylindole (DAPI) (blue). Scale bar, 50 μm. (P) Immunofluorescence (IF) analysis of nuclear factor of activated T cells, cytoplasmic 1 (NFATc1) nuclear translocation (green). Nuclei were stained with DAPI (blue). Scale bar, 5 μm. Data are presented as means ± SD (*n* = 5 per group). One-way ANOVA was used. Statistical significance: *P < 0.05, **P < 0.01, ***P < 0.001, and ****P < 0.0001; ns, not significant.

To investigate how DAB drives osteogenic processes, we stimulated BMSCs to differentiate into osteoblasts in osteogenic induction medium, either supplemented with DAB or left untreated, and quantitative real-time reverse transcription polymerase chain reaction (qRT-PCR) assays showed that DAB markedly increased the mRNA expression levels of the core osteogenic markers *RUNX2*, *OSX*, *ALP*, *COL1α1*, *OCN*, and *osteopontin* (*OPN*) (Fig. 2A). Western blot analyses further validated the pro-osteogenic activity of DAB, as the protein levels of these osteogenic markers were elevated in DAB-exposed BMSCs, with this up-regulation detectable as early as 5 d after differentiation initiation (Fig. 2B and Fig. S2A). Consistent with these gene and protein expression changes, ALP activity measurements revealed that ALP activity was greater in the DAB-treated groups than in the untreated control group (Fig. 2C), and subsequent ALP staining yielded results consistent with the activity assay findings (Fig. 2D and E). Moreover, alizarin red S (ARS) staining, which was used to evaluate calcium deposition—a marker of late-stage osteogenic differentiation and matrix mineralization—revealed that the DAB-treated group displayed substantially more extensive and intense positive staining, indicating robust promotion of mineralized nodule formation (Fig. 2F and G). These in vitro results illustrate that DAB effectively enhances the osteogenic differentiation of BMSCs by up-regulating the expression of key transcription factors and bone matrix proteins, which, in turn, accelerates the maturation and mineralization of the bone matrix.

To assess the therapeutic capacity of DAB in regulating osteoclastogenesis, we explored its impacts on RANKL-triggered osteoclast differentiation in mouse bone-marrow-derived macrophages (BMDMs). qRT-PCR analysis of osteoclastogenic markers revealed that DAB suppressed the mRNA expression of *cellular FBJ murine osteosarcoma viral oncogene homolog* (*c-Fos*), *nuclear factor of activated T cells*, *cytoplasmic 1* (*NFATc1*), *matrix metalloproteinase 9* (*MMP9*), *ACP5*, and *CTSK* in a concentration-dependent manner (Fig. 2H). Subsequent Western blot assays further confirmed that DAB down-regulated the protein expression of these markers (Fig. 2I and Fig. S2B), and this inhibitory effect on protein levels was also time dependent (Fig. 2J and Fig. S2C). In addition, the number and size of TRAP-positive multinucleated cells decreased with increasing DAB treatment dosage (Fig. 2K and L). Bone resorption assays using flat bovine femur bone slices further verified the inhibitory effect of DAB on osteoclast function (Fig. 2M and N). Rhodamine–phalloidin staining was used to evaluate the influence of DAB on cytoskeletal structure: Whereas the RANKL-only group exhibited intact actin rings, DAB treatment led to notable shrinkage of filamentous actin (F-actin) ring areas and a decrease in the number of fused osteoclast nuclei (Fig. 2O). Finally, immunofluorescence (IF) assays indicated that DAB strongly inhibited the RANKL-induced nuclear translocation of NFATc1 (Fig. 2P). Collectively, these findings reveal that DAB down-regulates the RANKL-mediated activation of NFATc1 and its downstream targets, thereby impairing osteoclast differentiation and attenuating bone resorption activity.

### DAB inhibits mTORC1 via the AMPK/TFEB–RPTOR axis to promote osteoblastogenesis

**Fig. 3.**
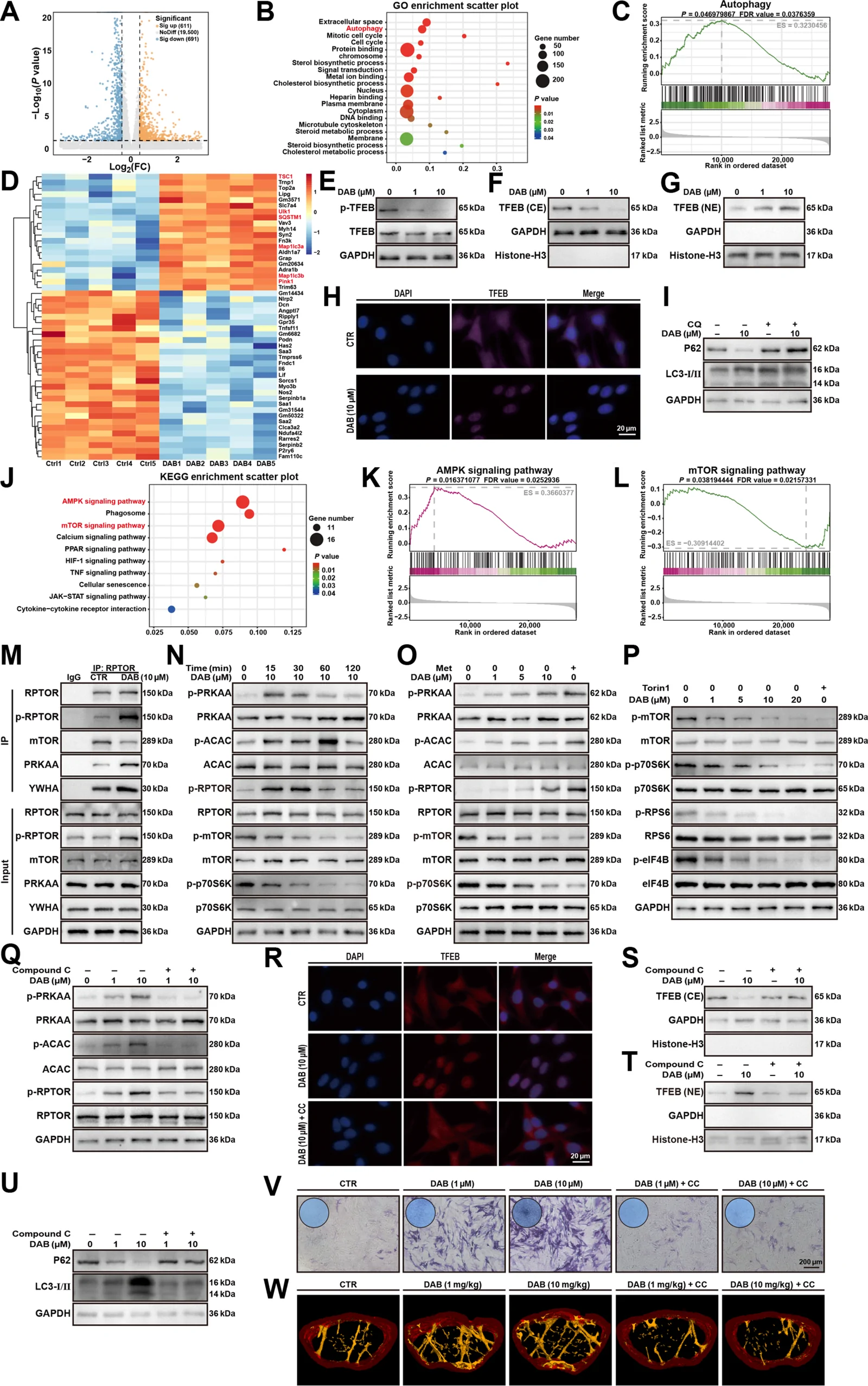
Dabigatran (DAB) inhibits mechanistic target of rapamycin complex 1 (mTORC1) via the adenosine-monophosphate-activated protein kinase (AMPK)/transcription factor EB (TFEB)–regulatory-associated protein of mTORC1 (RPTOR) axis to promote osteoblastogenesis. Transcriptomic profiling was performed in bone marrow mesenchymal stem cells (BMSCs) under osteogenic induction with or without DAB treatment. (A) Volcano plot illustrating the expression profiles of 20,802 genes identified by transcriptome sequencing. (B) Bubble plot of Gene Ontology (GO) enrichment results. (C) Gene set enrichment analysis (GSEA) results demonstrate up-regulation of the autophagy pathway following DAB treatment. (D) Heatmap showing changes in gene expression between the DAB-treated and control groups (*n* = 5 per group). (E) Western blot analysis of TFEB phosphorylation. (F and G) Western blot analysis of TFEB nuclear translocation. (H) Immunofluorescence (IF) analysis of TFEB nuclear translocation in BMSCs (purple). Nuclei were stained with 4′,6-diamidino-2-phenylindole (DAPI) (blue). Scale bar, 20 μm. (I) BMSCs were treated with DAB and chloroquine (CQ), and autophagy-related markers (p62 and microtubule-associated protein 1A/1B light chain 3 [LC3-I/II]) were analyzed by Western blotting. (J) Bubble plot of Kyoto Encyclopedia of Genes and Genomes (KEGG) enrichment analysis results. (K and L) GSEA results demonstrating up-regulation of the AMPK signaling pathway and down-regulation of the mTOR signaling pathway after DAB treatment. (M) Representative coimmunoprecipitation (Co-IP) assay for interactions between RPTOR and mTOR, protein kinase adenosine-monophosphate-activated catalytic subunit α (PRKAA), and tyrosine 3-monooxygenase/tryptophan 5-monooxygenase activation protein (YWHA) in BMSCs treated with or without 10 μM DAB. The phosphorylated form of RPTOR (p-RPTOR) in the immunoprecipitated complex was also detected. Immunoglobulin G (IgG) was used as a negative control for immunoprecipitation, and whole-cell lysates (input) were used as endogenous expression level controls. (N to P) BMSCs were treated with DAB at different time points and concentrations, and AMPK pathway activation was detected by Western blotting (metformin [Met] was used as the positive control, and torin1 served as the negative control). (Q) Western blot analysis demonstrated that compound C (CC) suppressed DAB-induced activation of the AMPK signaling pathway. (R) IF analysis showing that CC inhibits DAB-induced TFEB (red) nuclear translocation in BMSCs. Nuclei were stained with DAPI (blue). Scale bar, 20 μm. (S and T) Western blot analysis showing that CC inhibits TFEB nuclear translocation. (U) Western blot analysis showing that CC blocks DAB-induced up-regulation of autophagy-related markers. (V) Alkaline phosphatase (ALP) staining. Scale bar, 200 μm. (W) Representative micro-computed-tomography (CT) 3-dimensional (3D) reconstructions of the distal femur in aged mice. For in vivo experiments, data are presented as means ± SD (*n* = 6 per group). For in vitro experiments, data are presented as means ± SD (*n* = 5 per group). One-way ANOVA was used. ES, enrichment score; CE, cytoplasmic extract; NE, nuclear extract; FC, fold change; FDR, false discovery rate; PPAR, peroxisome-proliferator-activated receptor; HIF-1, hypoxia-inducible factor 1; JAK, Janus kinase; STAT, signal transducers and activators of transcription.

To identify the key downstream effectors through which DAB regulates bone metabolism, we performed transcriptomic analysis on primary mouse BMSCs treated with or without DAB. Compared with the control group, the DAB treatment group had 611 up-regulated genes and 691 down-regulated genes (Fig. 3A). Gene Ontology (GO) enrichment analysis revealed that pathways associated with autophagy were significantly enriched and up-regulated in the DAB-treated group compared with the control group (Fig. 3B). Furthermore, gene set enrichment analysis (GSEA) confirmed the enrichment of autophagy-related genes in DAB-treated BMSCs, which was consistent with the results of the GO analysis (Fig. 3C). A heatmap of autophagy-related genes showed distinct expression patterns between the DAB-treated and control groups, with most autophagy-related genes being differentially expressed upon DAB treatment (Fig. 3D). qRT-PCR further confirmed that DAB treatment induced the expression of autophagy-related genes (*sequestosome 1* [*SQSTM1*], *microtubule-associated protein 1 light chain 3 α* [*Map1lc3a*], *microtubule-associated protein 1 light chain 3 β* [*Map1lc3b*], *PTEN-induced kinase 1* [*Pink1*], *tuberous sclerosis complex 1* [*TSC1*], and *unc-51 like autophagy activating kinase 1* [*Ulk1*]) (Fig. S3A).

Autophagy is a cellular catabolic process that is dependent on the coordinated function of autophagosomes and lysosomes [25]. Transcription factor EB (TFEB), a master regulator of lysosomal biogenesis, coordinates this program by driving the expression of autophagy- and lysosome-related genes [26]. The nuclear localization and activity of TFEB are regulated by serine phosphorylation mediated by extracellular-signal-regulated kinase 2 and are modulated by extracellular nutrient levels [27]. Given the established role of TFEB in regulating autophagic activity, we investigated whether DAB activates TFEB in BMSCs. DAB treatment increased the nuclear localization of TFEB and reduced its phosphorylation at Ser211 (Fig. 3E to H and Fig. S3B). Using the lysosomal proteolysis inhibitor chloroquine (CQ), we found that DAB increased p62 degradation and promoted the formation of the microtubule-associated protein 1A/1B light chain 3 (LC3-I/II) in BMSCs (Fig. 3I and Fig. S3C). These results highlight the activation of TFEB-mediated autophagy by DAB in BMSCs.

Kyoto Encyclopedia of Genes and Genomes (KEGG) enrichment analysis and GSEA indicated that DAB activated the AMPK signaling pathway while suppressing the mTOR signaling pathway (Fig. 3J to L). Previous studies have shown that mTORC1 phosphorylates TFEB to promote its cytoplasmic retention, indicating that TFEB activity is primarily regulated by mTORC1 [28]. Phosphorylation of regulatory-associated protein of mTORC1 (RPTOR) by AMPK promotes its binding to tyrosine 3-monooxygenase/tryptophan 5-monooxygenase activation protein (YWHA), leading to the inhibition of mTORC1 kinase activity [29]. In this study, DAB treatment increased RPTOR phosphorylation, reduced the interaction between RPTOR and mTOR, and enhanced the association of RPTOR with protein kinase AMP-activated catalytic subunit α (PRKAA) and YWHA (Fig. 3M). Notably, DAB administration activated AMPK signaling, increased RPTOR phosphorylation, and decreased mTORC1 activity in BMSCs (Fig. 3N to P and Fig. S3D to F). These data demonstrate that DAB inhibits mTORC1 through the activation of AMPK.

Next, to elucidate whether DAB promotes osteogenic differentiation via AMPK activation, we treated cells with the AMPK inhibitor compound C (CC) (10 μM) under DAB treatment conditions. AMPK inhibition reduced the phosphorylation level of PRKAA, acetyl-coenzyme A carboxylase (ACAC), and RPTOR (Fig. 3Q and Fig. S3G). Concurrently, CC blocked the DAB-induced nuclear translocation of TFEB (Fig. 3R to T and Fig. S3H), p62 degradation, and LC3-I/II formation (Fig. 3U and Fig. S3I). In functional in vitro experiments, blockade of the AMPK signaling pathway by CC led to significant decreases in the mRNA expression levels of the osteogenic marker genes *RUNX2* and *OSX*, as detected by qRT-PCR (Fig. S3J), and ALP staining further indicated marked suppression of ALP activity (Fig. 3V and Fig. S3K). Furthermore, in in vivo experiments using aged mice treated with both DAB and CC, micro-CT analysis revealed that CC completely reversed the osteogenic-promoting effect of DAB (Fig. 3W and Fig. S3L), the groups were divided as follows: control (CTR), DAB (1 mg/kg), DAB (10 mg/kg), DAB (1 mg/kg) + CC, DAB (10 mg/kg) + CC. Collectively, these results demonstrate that activation of the AMPK–TFEB signaling pathway is essential for DAB to exert its osteogenic differentiation effects.

### DAB promotes osteoblastogenesis by targeting the PRKAB1 subunit to increase AMPK activity

**Fig. 4.**
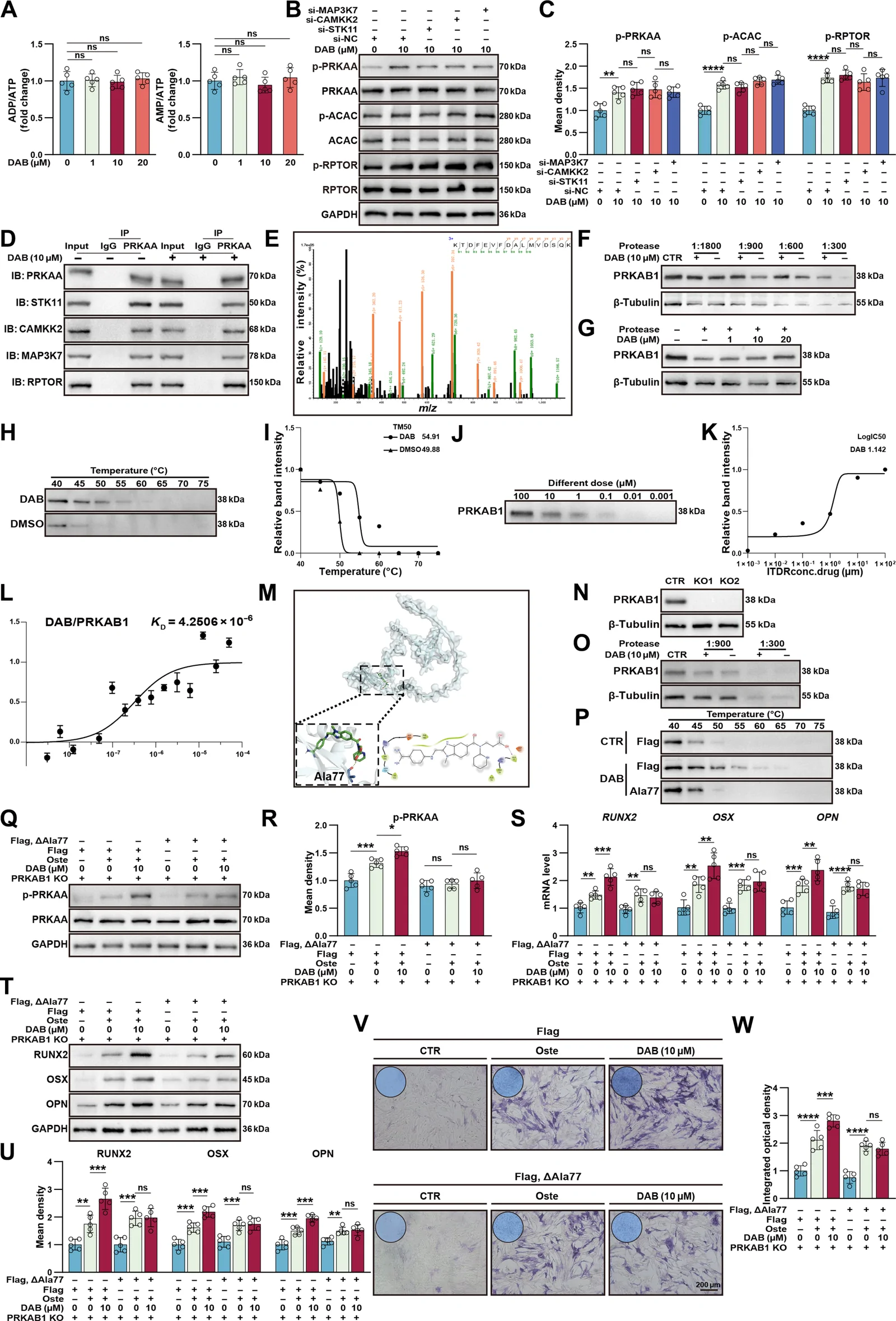
Dabigatran (DAB) promotes osteoblastogenesis by targeting the protein kinase adenosine monophosphate (AMP)-activated noncatalytic subunit β1 (PRKAB1) subunit to increase AMP-activated protein kinase (AMPK) activity. (A) Quantification of relative adenosine diphosphate (ADP)/adenosine triphosphate (ATP) and adenosine monophosphate (AMP)/ATP fold change in cells treated with 0, 1, 10, and 20 μM DAB for the indicated duration. (B and C) Western blot analysis of phosphorylated forms of protein kinase AMP-activated catalytic subunit α (p-PRKAA), acetyl-coenzyme A carboxylase (p-ACAC), and regulatory-associated protein of mTORC1 (p-RPTOR) in bone marrow mesenchymal stem cells (BMSCs) (B) after small interfering RNA (siRNA)-mediated knockdown of serine/threonine kinase 11 (STK11), calcium/calmodulin-dependent protein kinase kinase 2 (CAMKK2), or mitogen-activated protein kinase kinase kinase 7 (MAP3K7) and quantification (C). (D) Representative coimmunoprecipitation (Co-IP) assay showing the interactions of PRKAA with STK11, CAMKK2, MAP3K7, and RPTOR in BMSCs treated with or without 10 μM DAB. (E) Limited proteolysis-coupled mass spectrometry (LiP-MS) identification of PRKAB1 as a potential DAB target in BMSCs. (F and G) Drug affinity responsive target stability (DARTS) assay showing DAB-mediated protection of PRKAB1 from proteolysis. (H to K) Cellular thermal shift assay (CETSA) analysis: (H and I) Western blot of PRKAB1 after heat treatment with or without DAB, (J and K) Western blot of v-Rel reticuloendotheliosis viral oncogene homolog A (RELA) under different concentrations of DAB and isothermal dose-response fingerprinting-cellular thermal shift assay (ITDRF-CETSA) showing dose-dependent stabilization of PRKAB1 by DAB. (L) Interaction between DAB and PRKAB1 analyzed by microscale thermophoresis (MST) with a *K*_D_ of 4.2506 μM. (M) Molecular docking simulation of the interaction of DAB with human PRKAB1. (N) The knockout (KO) efficiency of PRKAB1 in MC3T3-E1 cells. (O to W) DARTS (O), CETSA (P), Western blot of phosphorylation of AMPK (Q) and quantification (R), quantitative real-time reverse transcription polymerase chain reaction (qRT-PCR) of osteogenic markers (*RUNX2*, *OSX*, and *OPN*) (S), Western blot of osteogenic markers (RUNX2, OSX, and OPN) (T) and quantification (U) , alkaline phosphatase (ALP) staining (scale bar, 200 μm) (V) and quantification (W) in Ala77 mutant or wild-type MC3T3-E1 cells. Data are presented as means ± SD (*n* = 5 per group). One-way ANOVA was used. Statistical significance: *P < 0.05, **P < 0.01, ***P < 0.001, and ****P < 0.0001; ns, not significant; IB, immunoblot; IC_50_, half-maximal inhibitory concentration.

AMPK activation is classically triggered by an increased AMP:adenosine triphosphate (ATP) or adenosine diphosphate (ADP):ATP ratio [30]. However, DAB treatment did not alter these cellular nucleotide ratios (Fig. 4A). We then examined whether DAB acts through known upstream kinases of AMPK, namely, serine/threonine kinase 11 (STK11), calcium/calmodulin-dependent protein kinase kinase 2 (CAMKK2), and mitogen-activated protein kinase kinase kinase 7 (MAP3K7). Knockdown of these kinases did not attenuate DAB-induced AMPK activation, as indicated by the sustained phosphorylation of PRKAA, ACAC, and RPTOR (Fig. 4B and C). Furthermore, DAB did not affect the interaction between PRKAA and these upstream regulators (Fig. 4D). Collectively, these data indicate that DAB enhances AMPK activity directly, independent of canonical energy-sensing pathways or upstream kinase stimulation.

To identify the direct protein target through which DAB activates AMPK and promotes osteogenesis, we used LiP-MS in mouse BMSCs. Proteomic analysis revealed the PRKAB1 subunit as a top candidate (Fig. 4E). Subsequent drug affinity responsive target stability (DARTS) assays followed by Western blotting confirmed that DAB protected PRKAB1 from proteolytic degradation in a dose-dependent manner (Fig. 4F and G). This interaction was further validated by a cellular thermal shift assay (CETSA), in which DAB treatment induced a dose-dependent shift in the thermal denaturation profile of PRKAB1, markedly stabilizing it against heat-induced aggregation, particularly at 50 °C. Melting curve analysis revealed a marked increase in the melting temperature from 49.88 °C in the controls to 54.91 °C in the DAB-treated samples. A fixed-temperature CETSA (50 °C) with increasing DAB concentration yielded an estimated half-maximal effective concentration (EC_50_) of 1.142 μM (Fig. 4H to K). In addition, MST (microscale thermophoresis) demonstrated high-affinity binding between recombinant PRKAB1 and DAB, with a *K*_D_ of 4.2506 μM (Fig. 4L). Specificity was confirmed, as DARTS and CETSA analyses revealed no interaction between DAB and other AMPK subunits (Fig. S4A and B). Molecular docking revealed the detailed binding mode: DAB forms a key 2.1-Å hydrogen bond with Ala77 of PRKAB1, supported by hydrophobic contacts (Ala73, Ala77, Pro79, Ile153, Val155, and Phe160), a polar interaction with Gln154, and electrostatic interactions with Asp117, Arg78, Lys156, and Asp159 (Fig. 4M).

To establish the functional necessity of PRKAB1 in DAB-mediated osteogenesis, we generated a PRKAB1-knockout MC3T3-E1 cell line using clustered regularly interspaced short palindromic repeats and CRISPR-associated protein 9 (CRISPR–Cas9) (Fig. 4N) and rescued it with either wild-type or Ala77-mutant PRKAB1. CETSA and DARTS confirmed that the Ala77 mutation abolished the DAB–PRKAB1 interaction (Fig. 4O and P). Moreover, this mutation prevented DAB from inducing AMPK phosphorylation (Fig. 4Q and R). In knockout cells reconstituted with the mutant, DAB failed to induce the mRNA and protein expression of osteogenic markers (*RUNX2*, *OSX*, and *OPN*) or increase ALP activity, as shown by qRT-PCR (Fig. 4S), Western blotting (Fig. 4T and U), and ALP staining (Fig. 4V and W). Similarly, knockdown of PRKAB1 in BMSCs significantly attenuated the pro-osteogenic effects of DAB (Fig. S5A to F). Mutation of Ala77 did not compromise basal AMPK pathway integrity, as the responses to the activator 5-aminoimidazole-4-carboxamide ribonucleotide (AICAR) and the inhibitor CC remained intact (Fig. S6A and B). However, in mutant-reconstituted BMSCs, DAB no longer modulated AMPK/mTORC1 activity or the transcriptional output of TFEB (Fig. S6C and D).

In summary, these results demonstrate that DAB directly binds to the Ala77 residue of the PRKAB1 subunit, leading to allosteric activation of the AMPK signaling pathway. This specific molecular interaction is strictly required for DAB to promote osteogenic differentiation.

### DAB inhibits osteoclastogenesis via the AMPK/NF-κB signaling pathway

**Fig. 5.**
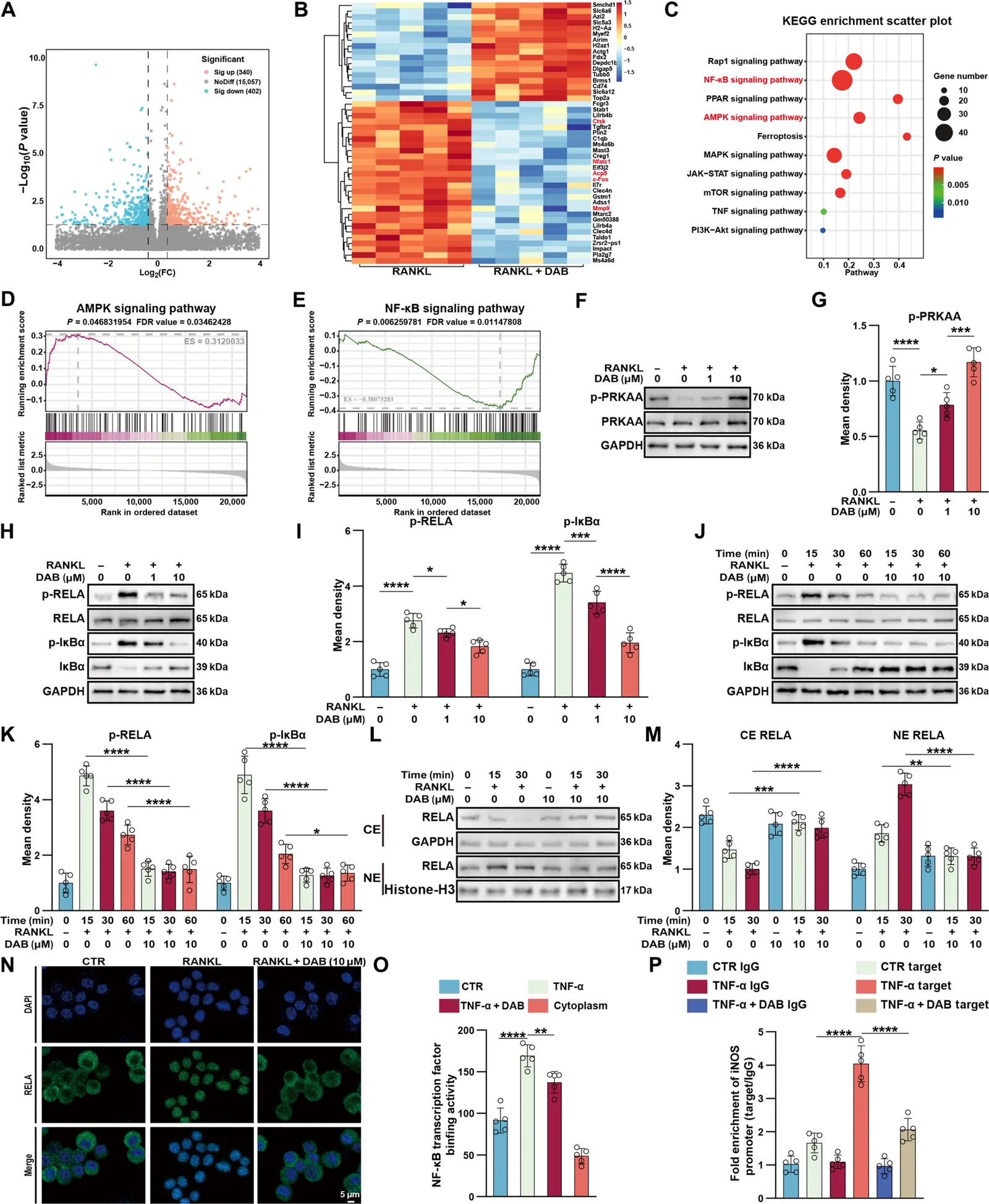
Dabigatran (DAB) inhibits osteoclastogenesis via the adenosine-monophosphate-activated protein kinase (AMPK)/nuclear factor κB (NF-κB) signaling pathway. Transcriptomic profiling was performed in receptor activator of NF-κB ligand (RANKL)-stimulated bone-marrow-derived macrophages (BMDMs) with or without DAB treatment. (A) The volcano plot illustrates the expression profiles of 15,799 genes identified by transcriptome sequencing. (B) Heatmap showing changes in gene expression between the DAB-treated and control groups (*n* = 5 per group). (C) Bubble plot of Kyoto Encyclopedia of Genes and Genomes (KEGG) enrichment analysis results. (D and E) Gene set enrichment analysis (GSEA) results demonstrate up-regulation of the AMPK pathway (D) and down-regulation of the NF-κB pathway (E) following DAB treatment. (F and G) Western blot of phosphorylation of AMPK in BMDMs treated with different concentrations of DAB under RANKL stimulation (F) and quantification (G). (H and I) Western blot analysis of phosphorylation of v-Rel reticuloendotheliosis viral oncogene homolog A (RELA) and inhibitor of NF-κBα (IκBα) in BMDMs treated with different concentrations of DAB under RANKL stimulation (H) and quantification (I). (J and K) Western blot analysis of phosphorylation of RELA and IκBα in BMDMs treated with DAB for different time points under RANKL stimulation (J) and quantification (K). (L and M) Western blot analysis of RELA nuclear translocation in BMDMs treated with DAB for different time points under RANKL stimulation (L) and quantification (M). (N) Immunofluorescence (IF) assay for RELA (green) nuclear translocation in BMDMs; nuclei were stained with 4′,6-diamidino-2-phenylindole (DAPI) (blue). Scale bar, 5 μm. (O) NF-κB transcription factor binding activity was measured in control (CTR), tumor necrosis factor-α (TNF-α), TNF-α + DAB, and cytoplasm groups. (P) Chromatin immunoprecipitation (ChIP) assay was performed to analyze the fold enrichment of inducible nitric oxide synthase (iNOS) promoter in CTR, TNF-α, TNF-α + DAB groups, with immunoglobulin G (IgG) fractions as negative controls. Data are presented as means ± SD (*n* = 5 per group). One-way ANOVA was used. Statistical significance: *P < 0.05, **P < 0.01, ***P < 0.001, and ****P < 0.0001; ns, not significant; MAPK, mitogen-activated protein kinase; PI3K, phosphatidylinositol 3-kinase.

To elucidate the mechanism underlying the DAB-mediated inhibition of osteoclast differentiation, we performed RNA sequencing on RANKL-stimulated BMDMs treated with or without DAB. Transcriptomic analysis revealed significant alterations in gene expression, with 340 genes up-regulated and 402 genes down-regulated upon DAB treatment (Fig. 5A). A heatmap was constructed, which illustrated the marked suppression of osteoclast-specific genes in the DAB-treated group (Fig. 5B). KEGG enrichment and GSEA further revealed significant enrichment of genes associated with the AMPK and NF-κB signaling pathways, suggesting their involvement in the antiosteoclastogenic effect of DAB (Fig. 5C to E).

To investigate these pathways further, we examined the activation status of AMPK and NF-κB. Western blot analysis revealed that DAB treatment increased the phosphorylation of PRKAA while simultaneously attenuating the RANKL-induced phosphorylation of RELA and the degradation of inhibitor of NF-κBα (IκBα) (Fig. 5F to K). Given that NF-κB is a classical signaling pathway that regulates osteoclastogenesis and that nuclear translocation and subsequent DNA binding are critical steps in its activation, we next assessed the effect of DAB on these processes. Subcellular fractionation confirmed that DAB prevented the nuclear translocation of the RELA subunit (Fig. 5L to N). Furthermore, we evaluated the impact of DAB on the DNA binding activity of nuclear NF-κB. Intriguingly, DAB significantly impaired NF-κB DNA binding activity in BMDMs (Fig. 5O). Given that NF-κB-mediated chronic low-grade inflammation promotes osteoclast differentiation, we performed chromatin immunoprecipitation (ChIP)–PCR to examine the binding of RELA to the promoter regions of inducible nitric oxide synthase (iNOS). The results revealed that DAB disrupted the association of RELA with these promoters (Fig. 5P). Collectively, these data indicate that DAB inhibits osteoclast differentiation, likely by interfering with the DNA binding capacity of RELA to the promoters of osteoclastogenic genes. Taken together, our results demonstrate that DAB concurrently activates AMPK signaling and suppresses the NF-κB pathway during osteoclastogenesis.

Given our prior finding that DAB activates AMPK in osteoblasts by directly binding to the PRKAB1 subunit, we investigated whether its antiosteoclastogenic effect similarly depends on PRKAB1. Pharmacological inhibition of AMPK using CC partially attenuated the DAB-mediated suppression of osteoclast marker expression (Fig. S7A to C). Furthermore, after the endogenous expression of PRKAB1 in BMDMs was down-regulated by small interfering RNA (siRNA), the cells were transfected with either wild-type or mutant PRKAB1. The results revealed that the inhibitory effect of DAB on osteoclast differentiation was partially attenuated (Fig. S7D to F). Together, these data demonstrate that DAB inhibits osteoclastogenesis in a manner partially dependent on its interaction with PRKAB1, suggesting that additional, yet-to-be-identified targets contribute to its full antiresorptive activity.

### RELA is a key target of DAB for inhibiting osteoclastogenesis

**Fig. 6.**
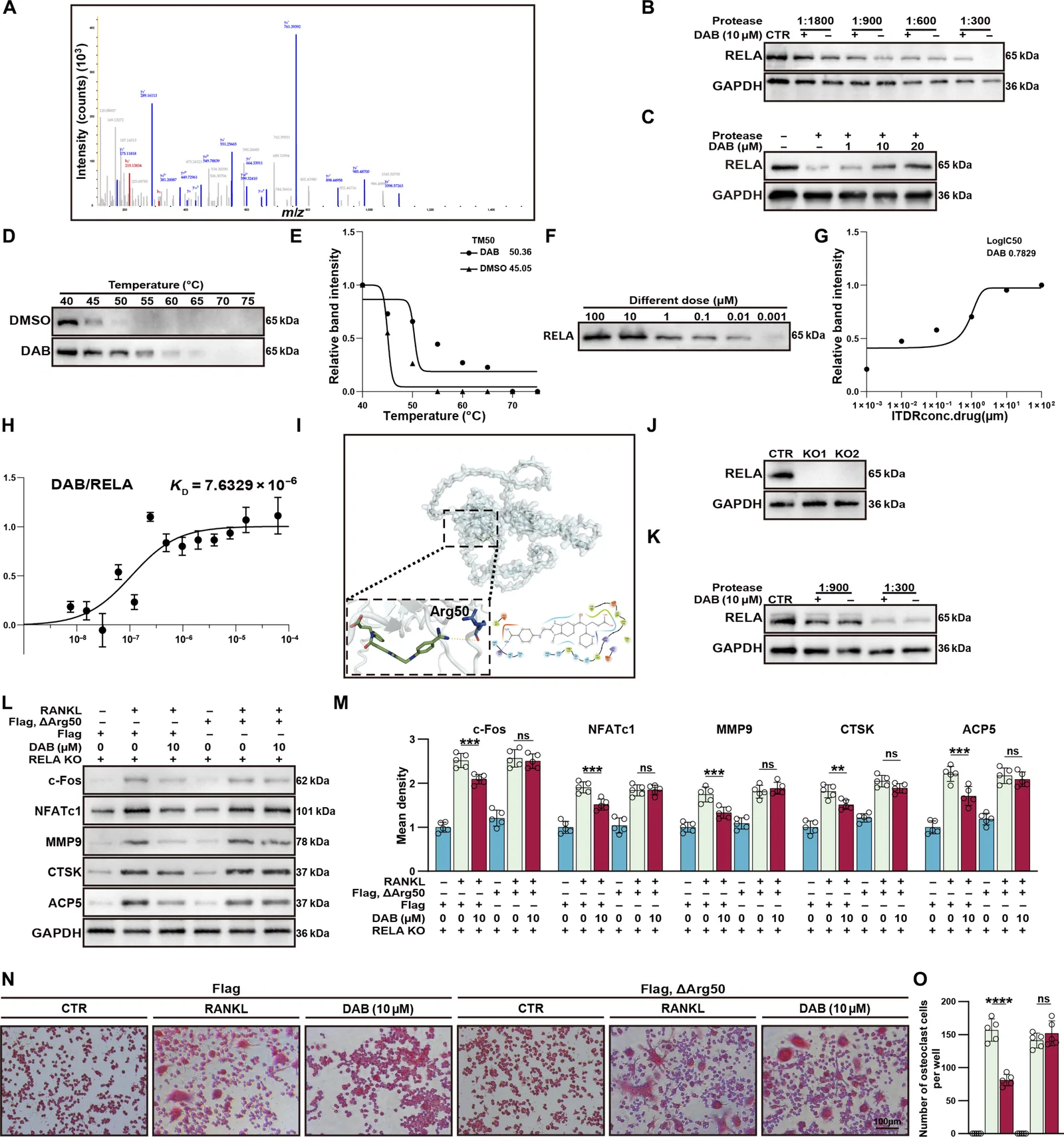
v-Rel reticuloendotheliosis viral oncogene homolog A (RELA) is a key target of dabigatran (DAB) for inhibiting osteoclastogenesis. (A) Limited proteolysis-coupled mass spectrometry (LiP-MS) identification of RELA as a potential DAB target in bone-marrow-derived macrophages (BMDMs). (B and C) Drug affinity responsive target stability (DARTS) assay showing DAB-mediated protection of RELA from proteolysis. (D to G) cellular thermal shift assay (CETSA) analysis: (D and E) Western blot of RELA after heat treatment with or without DAB; (F and G) Western blot of RELA under different concentrations of DAB and isothermal dose-response fingerprinting-cellular thermal shift assay (ITDRF-CETSA) showing dose-dependent stabilization of RELA by DAB. (H) Microscale thermophoresis (MST) measurement of the binding affinity between DAB and recombinant RELA protein, with a *K*_D_ of 7.6329 μM. (I) Molecular docking simulation of the interaction of DAB with human RELA. (J) Western blot validation of RELA knockout (KO) and reconstitution in RAW264.7 cells. (K to O) DARTS (K), Western blot of osteoclastogenic markers (cellular FBJ murine osteosarcoma viral oncogene homolog [c-Fos], nuclear factor of activated T cells, cytoplasmic 1 [NFATc1], matrix metalloproteinase 9 [MMP9], acid phosphatase 5 [ACP5], and cathepsin K [CTSK]) (L) and quantification (M), tartrate-resistant acid phosphatase (TRAP) staining (scale bar, 100 μm) (N) and quantification of the number and area of TRAP-positive cells (O) in Arg50 mutant or wild-type RAW264.7 cells. Data are presented as means ± SD (*n* = 5 per group). One-way ANOVA was used. Statistical significance: **P < 0.01, ***P < 0.001, and ****P < 0.0001; ns, not significant.

To identify the key protein target through which DAB suppresses osteoclastogenesis, we performed DARTS analysis using BMDM lysates. Proteins were separated by sodium dodecyl sulfate-polyacrylamide gel electrophoresis (SDS-PAGE), and the band specifically protected by DAB from proteolysis was identified by MS as the NF-κB subunit RELA (Fig. 6A). Follow-up DARTS assays with a titration of protease concentrations confirmed that DAB protected RELA from enzymatic degradation in a dose-dependent manner (Fig. 6B and C). This direct interaction was further validated by CETSA, in which DAB treatment stabilized RELA against heat-induced denaturation in a concentration-dependent manner (Fig. 6D to G). In addition, MST confirmed high-affinity, direct binding between DAB and recombinant RELA protein, with a *K*_D_ of 7.6329 μM (Fig. 6H).

To further elucidate the interaction mode between DAB and RELA, we performed molecular docking simulations. The docking results revealed that DAB forms a key hydrogen bond (distance, 2.8 Å) with the Arg50 residue of RELA (Fig. 6I). In addition, DAB engages in multiple interactions with surrounding amino acids, including hydrophobic interactions with Tyr100, Leu104, Cys105, Val72, Ile110, and Phe113 and polar interactions with His111, Ser112, Gln114, Asn115, Thr57, His58, and Thr60. These collective interactions stabilize the DAB–RELA complex. To identify the key amino acid residues for RELA–DAB binding, we generated a RELA-knockout RAW264.7 cell line using CRISPR–Cas9 and rescued it with plasmids expressing either wild-type or mutant RELA (Fig. 6J). The binding of DAB to different RELA variants was subsequently assessed using DARTS. The results showed that DAB conferred clear protease protection and thermal stabilization to wild-type RELA, but this protective effect was completely absent for the Arg50 mutant (Fig. 6K). These findings confirm that Arg50 is the key amino acid residue required for DAB binding and the subsequent functional regulation of RELA.

To validate the functional significance of Arg50, we generated a RELA-knockout RAW264.7 cell line using CRISPR–Cas9 and rescued it with either wild-type RELA or an Arg50 mutant. Functionally, whereas the Arg50 mutation did not impair the basal responsiveness of the NF-κB pathway to an activator such as tumor necrosis factor-α (TNF-α) and the inhibitory effect of DAB on the NF-κB pathway is dependent on the Arg50 residue (Fig. S8A to E), it completely abrogated the ability of DAB to suppress osteoclast differentiation, as evidenced by Western blotting and TRAP staining (Fig. 6L to O). Similarly, siRNA-mediated knockdown of RELA in BMDMs eliminated the inhibitory effect of DAB on the mRNA and protein expression of osteoclastogenic markers, which was further corroborated by TRAP staining (Fig. S8F to S).

Collectively, these findings demonstrate that RELA is a direct functional target of DAB and that the interaction of RELA with Arg50 is essential for the inhibition of the NF-κB pathway and suppression of osteoclast differentiation by DAB.

## Discussion

Osteoporosis is a systemic skeletal disorder caused by an imbalance between bone formation and bone resorption [31,32]. The development of safe and cost-effective therapies capable of simultaneously and bidirectionally regulating bone homeostasis represents a substantial unmet clinical need [33,34]. This study is the first to report that the oral anticoagulant DAB markedly promotes osteogenesis and inhibits osteoclastogenesis in both in vitro and in vivo models. More importantly, through an integrated approach combining chemical proteomics, structural biology, and functional validation, we elucidated a novel “single-molecule, dual-target” mechanism: DAB directly binds to and activates the AMPK subunit PRKAB1 to promote osteogenic differentiation while concurrently binding to and inhibiting the key NF-κB subunit RELA to block osteoclast differentiation. This elucidation not only provides a solid theoretical foundation for repositioning DAB as a candidate antiosteoporotic agent but also offers a new strategic paradigm for treating metabolic bone diseases.

### Discovery of a bidirectional bone homeostasis modulator and the value of drug repurposing

Currently, the mechanisms of action of first-line clinical antiosteoporosis drugs are relatively singular and are primarily categorized into inhibitors of bone resorption (e.g., bisphosphonates) or promoters of bone formation (e.g., teriparatide) [35–37]. The long-term use of these drugs is often limited by specific adverse effects, high economic costs, or the need for injection administration, which compromises patient compliance and long-term clinical benefits [38]. To address this unmet clinical need, we conducted a phenotype-driven screening of FDA-approved drugs using dual indicators central to bone remodeling—the osteogenic factor RUNX2 and the osteoclastic factor NF-κB. This screening identified the direct oral thrombin inhibitor DAB as a novel candidate drug with dual-direction regulatory effects.

Notably, previous clinical observational studies have provided preliminary but vague clues linking DAB to skeletal health. A retrospective cohort study in patients with nonvalvular atrial fibrillation revealed that compared with warfarin use, DAB use was associated with a lower risk of osteoporotic fractures [39]. However, that study failed to elucidate the underlying biological mechanism.

Our study clearly reveals the direct pharmacological effects of DAB within the skeletal system. In vitro experiments confirmed that DAB not only directly promotes the osteogenic differentiation of BMSCs but also effectively inhibits RANKL-induced osteoclastogenesis. In vivo studies further validated its bone-protective effects: In both an OVX mouse model mimicking postmenopausal osteoporosis and a naturally aged mouse model mimicking senile osteoporosis, oral administration of DAB markedly alleviated bone loss and preserved trabecular microarchitecture. These robust preclinical data, combined with the well-defined bioavailability, pharmacokinetic profile, and safety record of DAB accumulated through long-term clinical use, fully demonstrate the unique advantage of the drug repurposing strategy—bypassing the lengthy and costly early stages of drug development, it holds promise for rapidly translating a fundamental discovery into a new clinical therapeutic strategy for chronic diseases such as osteoporosis.

### Molecular mechanism of the osteogenic effect of DAB

Autophagy, a highly conserved lysosomal degradation pathway, plays a critical role in maintaining BMSC stemness, promoting osteogenic differentiation, and protecting osteoblasts from apoptosis and senescence, particularly under pathological conditions such as aging and estrogen deficiency [40,41]. Substantial evidence indicates that impaired autophagic flux in BMSCs is a key driver of age-related and postmenopausal bone loss and that enhancing autophagy effectively restores bone formation capacity [42]. In this study, we demonstrate for the first time that DAB markedly promotes autophagosome formation, enhances lysosomal function, and increases TFEB-mediated autophagic flux in BMSCs, which constitute the core cellular mechanism underlying its osteogenic effect.

At the molecular level, using LiP-MS, an advanced chemoproteomic technique that unbiasedly identifies drug–target interactions in a native cellular environment, we successfully identified PRKAB1 as a direct target of DAB in BMSCs. AMPK, a master regulator of cellular energy homeostasis, has recently emerged as a key target for metabolic bone disease therapy [43]. However, most previously reported AMPK agonists (e.g., metformin [Met] and AICAR) act indirectly, primarily by altering the intracellular AMP/ATP ratio or targeting upstream kinases, which generally lack subunit specificity and are prone to off-target effects [44,45]. In contrast, our series of biophysical and functional assays (including DARTS, CETSA, MST, and site-directed mutagenesis) consistently confirmed that DAB directly binds to Ala77 within the carbohydrate-binding module of the PRKAB1 subunit, thereby allosterically activating the AMPK holoenzyme independent of changes in cellular energy status. To our knowledge, this is the first report of a small molecule activating AMPK signaling in the skeletal system by directly targeting PRKAB1.

Activated AMPK subsequently phosphorylates RPTOR, a key component of the mTORC1 complex, promoting its sequestration in the cytoplasm by binding to YWHA, thereby inhibiting mTORC1 kinase activity. The inhibition of mTORC1, a master negative regulator of autophagy, leads to the dephosphorylation and nuclear translocation of its downstream substrate TFEB [46–48]. Nuclear TFEB initiates the transcription of a suite of autophagy- and lysosome-related genes, ultimately enhancing autophagic flux and driving osteogenic differentiation in BMSCs [49]. Crucially, knockdown of PRKAB1 or site-directed mutation of the binding site Ala77 completely abolished DAB-induced AMPK activation and enhanced autophagy and osteogenic effects. These findings precisely confirm that PRKAB1 is an indispensable direct target mediating the anabolic effects of DAB in BMSCs. This discovery not only elucidates a novel mechanism of small-molecule AMPK activation but also provides a new pharmacological paradigm for developing subunit-specific AMPK agonists for osteoporosis treatment.

### Molecular mechanism of the antiosteoclastic activity of DAB

The NF-κB signaling pathway is the central regulatory hub for RANKL-induced osteoclast differentiation, activation, and survival. Its hyperactivation is a key pathological mechanism underlying increased bone resorption in primary osteoporosis [50,51]. However, given the broad physiological functions of NF-κB in immune responses and the survival of various cell types, the development of clinically applicable NF-κB inhibitors has long been hampered by marked off-target effects and systemic toxicity [52].

In this study, we found that DAB effectively inhibited the RANKL-induced NF-κB activation cascade, including the phosphorylation and degradation of IκBα, the phosphorylation of RELA at Ser536, and the subsequent nuclear translocation of RELA. More importantly, ChIP assays further revealed that DAB significantly attenuated the DNA binding capacity of nuclear RELA and its recruitment to the promoter regions of osteoclastogenesis-related genes (e.g., NFATc1 and CTSK), which is the rate-limiting step for the downstream transmission of NF-κB signaling to initiate osteoclast differentiation.

Through chemoproteomic screening, we identified RELA, the core subunit of the canonical NF-κB pathway, as the direct binding target of DAB in osteoclast precursor cells. Molecular docking simulations and site-directed mutagenesis validation demonstrated that DAB directly binds to Arg50 within the N-terminal Rel homology domain of the RELA protein via stable hydrogen bonds and hydrophobic interactions. The Rel homology domain is responsible for the DNA binding, dimerization, and nuclear translocation of RELA [53]. Arg50 is a key amino acid that directly mediates the interaction of RELA with the κB consensus sequence in target gene promoters. Consistent with these findings, mutation of Arg50 completely abolished the inhibitory effects of DAB on both NF-κB transcriptional activity and osteoclast differentiation, confirming that this direct binding is the molecular basis for the antiresorptive activity of DAB.

Notably, although we observed that DAB could also activate AMPK signaling in osteoclast precursor cells, potentially contributing to its antiosteoclastic effect, genetic blockade of RELA signaling was sufficient to completely abolish the inhibitory effect of DAB on osteoclast formation. These findings indicate that direct targeting of RELA is the core and independent mechanism of its antiresorptive activity. Unlike traditional NF-κB inhibitors that mostly target upstream kinases and indirectly inhibit the pathway, DAB directly targets the RELA subunit and blocks its DNA binding capacity, representing a more specific and potent mode of NF-κB inhibition. These findings not only elucidate the molecular mechanism underlying the antiresorptive effect of DAB but also provide a new structural basis and theoretical foundation for the development of transcription-factor-targeted NF-κB inhibitors with potentially increased tissue specificity and safety.

### Limitations and future perspectives

Although this study provides robust preclinical evidence and novel mechanistic insights, its limitations must be acknowledged. First, all in vivo efficacy data were derived from mouse models of osteoporosis; the extrapolation of these findings to human patients requires cautious validation. The optimal dose, dosing regimen, and long-term efficacy of DAB for treating human osteoporosis need to be confirmed in well-designed prospective clinical trials. Second, as a direct oral anticoagulant, the potential bleeding risk associated with long-term DAB use in patients with nonthrombotic osteoporosis represents a critical safety concern that requires focused evaluation during clinical translation. While the RE-LY (randomized evaluation of long-term anticoagulant therapy) trial and subsequent real-world studies have established that DAB carries a lower overall bleeding risk compared to warfarin in anticoagulation-eligible populations, these data were derived exclusively from patients with established thrombotic indications [54]. Notably, the present study was designed with a primary focus on skeletal phenotypes and did not include comprehensive coagulation function assessments (e.g., activated partial thromboplastin time and prothrombin time) during the 60-d treatment period. Consequently, subclinical alterations in hemostatic parameters cannot be definitively excluded. The bleeding risk profile in patients with nonthrombotic osteoporosis who may differ substantially in baseline bleeding risk, comorbidity patterns, and risk–benefit calculus remains entirely uncharacterized. Furthermore, the elderly population predominantly affected by osteoporosis often presents with age-related renal impairment, polypharmacy, and increased fall propensity, all of which may synergistically amplify bleeding susceptibility. Therefore, dedicated prospective studies are warranted to delineate the safety boundary of DAB repurposing in this novel clinical context.

Future research could focus on several directions: (a) conducting dedicated toxicological studies and carefully designed phase I clinical trials to rigorously delineate the safety boundary of DAB in a nonthrombotic osteoporosis population and to establish an appropriate risk–benefit assessment framework prior to large-scale efficacy testing; (b) conducting multicenter, randomized, double-blind, placebo-controlled phase II/III clinical trials to definitively assess the efficacy and safety of DAB in treating postmenopausal and senile osteoporosis; (c) using medicinal chemistry approaches to designing and synthesizing bone-targeted DAB derivatives or novel analogs with anticoagulant activity separated from bone-protective effects, aiming to further optimize efficacy and safety; (d) investigating the effects of DAB on the interactions between multiple cell types and on immune regulation within the bone marrow microenvironment; (e) evaluating the therapeutic potential of DAB in other skeletal disorders characterized by imbalanced bone homeostasis, such as delayed fracture healing, rheumatoid-arthritis-associated bone erosion, diabetic osteoporosis, and glucocorticoid-induced osteoporosis; and (f) exploring whether the “single-molecule, dual-target” pharmacological paradigm revealed in this study could provide new insights and strategies for developing innovative therapies for other metabolic diseases.

## Conclusion

In summary, through phenotype-based drug repurposing screening, systematic preclinical validation, and in-depth mechanistic exploration, this study successfully repurposes the widely used oral anticoagulant DAB as a novel dual-direction modulator of bone homeostasis. We demonstrate for the first time that DAB exerts bone-protective effects via a dual mechanism independent of its anticoagulant activity: In BMSCs, DAB directly binds to Ala77 of PRKAB1, activating the AMPK/mTORC1/autophagy axis to promote osteogenic differentiation; in osteoclast precursors, DAB targets Arg50 of RELA, blocking NF-κB transcriptional activity to inhibit osteoclastogenesis. These findings elucidate the direct molecular targets and cell-type-specific mode of action of DAB, confirming that its skeletal benefits are independent of its anticoagulant effects and providing a mechanistic basis for its dual regulation of bone homeostasis. This unique “single-molecule, dual-target” mode of action enables DAB to synergistically restore the balance between bone formation and bone resorption, thereby effectively alleviating bone loss in mouse models of postmenopausal and senile osteoporosis. To facilitate clinical translation, real-world studies and prospective trials are warranted to validate the bone-protective efficacy of DAB in osteoporotic populations and to define the optimal dosing window that balances skeletal benefits against bleeding risk. From a drug development perspective, the identified dual-target binding mode can guide the structural optimization of DAB derivatives to enhance bone tissue selectivity and minimize anticoagulant side effects, thereby offering a novel strategy for designing multifunctional antiosteoporosis agents. This work not only identifies a highly promising oral bifunctional drug candidate for the prevention and treatment of osteoporosis but also establishes a new pharmacological paradigm for skeletal disease therapy, offering new insights and strategies for both mechanistic research and clinical intervention in metabolic bone diseases.

## Materials and Methods

### Animals

The animal research protocols used were examined and sanctioned by the Medical Ethics Committee of Qilu Hospital, Shandong University, bearing approval reference KYLL-2024(ZM)-1243.

For the in vivo safety evaluation, 18 C57BL/6J mice (9 males and 9 females) were randomly assigned to 3 groups. The mice received daily oral gavage of DAB at various doses for 60 consecutive days, while the control group was given an equivalent volume of vehicle (F-12 medium containing DMSO). All mice were euthanized at the end of the 60-d treatment period.

To investigate the inhibitory effect of DAB on osteoclasts and its osteogenic promotion in vivo, a collective of 42 C57BL/6J mice were utilized, including 24 females at 12 weeks of age and 18 aged males of 24 months. Mice were assigned in a random manner to experimental sets, each containing 6 animals, so as to guarantee group homogeneity. Surgical interventions for the 12-week-old female mice, which included OVX or sham control procedures, were performed under general anesthesia; analgesics were subsequently provided as necessary to manage any postoperative discomfort [55]. The aged male mice started receiving drug intervention at 24 months of age. All mice were euthanized upon completion of the 60-d treatment period.

To determine whether DAB promotes osteogenesis in vivo through the AMPK signaling pathway, 30 aged male C57BL/6J mice were randomly divided into 5 groups. Mice received daily oral gavage of DAB at various doses, alone or in combination with CC (20 mg/kg), for 60 consecutive days, while the control group was given an equivalent volume of vehicle. All mice were euthanized at the end of the 60-d treatment period.

Throughout the study, animals were maintained at a specific-pathogen-free facility of Shandong University under rigorously controlled conditions—temperature was kept at 22 ± 1 °C, humidity was maintained at 55 ± 10%, and lighting was scheduled on a 12-h light/12-h dark rotation. Sterilized feed and autoclaved drinking water were accessible without restriction during both the habituation and study phases. Every animal-based experimental procedure adhered to the recommendations set forth in the National Institutes of Health Guide for the Care and Use of Laboratory Animals and was reported consistent with the Animal Research: Reporting of In Vivo Experiments guidelines.

### Antibodies and reagents

Unless otherwise indicated, all primary antibodies were diluted at 1:1,000. Antibodies targeting RUNX2, OSX, ALP, COL1α1, OCN, OPN, c-Fos, NFATc1, MMP9, CTSK, ACP5, TFEB, p-TFEB, histone-H3, p62, LC3-I/II, RPTOR, p-RPTOR, mTOR, p-mTOR, PRKAA, p-PRKAA, YWHA, ACAC, p-ACAC, 70-kDa ribosomal protein S6 kinase (p70S6K), p-p70S6K, ribosomal protein S6 (RPS6), p-RPS6, eukaryotic translation initiation factor 4B (eIF4B), p-eIF4B, MAP3K7, CAMKK2, STK11, RELA, IκBα, p-IκBα, cyclooxygenase 2 (COX2), iNOS, and PRKAG2, as well as normal rabbit and mouse immunoglobulin G (IgG) isotype controls for ChIP and coimmunoprecipitation (Co-IP) assays, were purchased from Cell Signaling Technology (Boston, MA, USA). Antibodies against PRKAA1, PRKAA2, PRKAB1, PRKAB2, PRKAG1, and PRKAG3 were obtained from ABclonal Biotechnology (Wuhan, China). Anti-β-tubulin (1:10,000) and anti-green fluorescent protein (GFP; 1:1,000) antibodies were supplied by Abways Technology (Shanghai, China); anti-GFP antibody was utilized to verify cellular transfection efficiency. Anti-glyceraldehyde-3-phosphate dehydrogenase (GAPDH) antibody (1:10,000), horseradish peroxidase (HRP)-conjugated goat anti-rabbit and anti-mouse IgG secondary antibodies (1:10,000), and the PV-9000 IHC kit containing biotin-conjugated secondary antibody and streptavidin–HRP were acquired from ZSGB-BIO (Beijing, China). For IF staining, fluorescein isothiocyanate (FITC)- or rhodamine-labeled goat anti-rabbit IgG secondary antibodies were diluted to 1:500 for incubation. The IHC staining kit was purchased from Zhongshan Golden Bridge Biotechnology (Beijing, China). Calcein, CQ, and Met were obtained from Sigma-Aldrich (St. Louis, MO, USA). Torin 1, AICAR, and CC were supplied by MedChemExpress (Monmouth Junction, NJ, USA). Recombinant murine macrophage colony-stimulating factor (M-CSF), recombinant mouse RANKL, and recombinant mouse TNF-α were purchased from R&D Systems (Minneapolis, MN, USA). Sodium β-glycerophosphate, ascorbic acid, ARS solution, and dexamethasone were sourced from Solarbio (Beijing, China). The CCK-8 assay kit and ELISA kits for P1NP, OCN, ACP5, and CTSK were procured from Elabscience Biological Technology (Wuhan, China). Staining kits for ALP and ALP activity quantification kit were purchased from Beyotime (Shanghai, China). TRAP staining kit were purchased from Servicebio (Wuhan, China). All small-molecule compounds were dissolved in DMSO to prepare stock solutions, and recombinant cytokines were reconstituted following the manufacturer’s standard protocols.

### In vitro experiments

#### Cell culture and differentiation

BMDMs were harvested from C57BL/6 mice following euthanasia via cervical dislocation, after which the tibiae and femurs were excised aseptically. After both epiphyses were cut open, the bone marrow cavity was flushed using a 1-ml syringe loaded with high-glucose Dulbecco’s modified Eagle’s medium (DMEM) containing 10% fetal bovine serum (FBS) until the bone shafts turned white. The harvested marrow cells were plated into 6-well dishes and maintained for 6 d in complete medium supplemented with M-CSF (10 ng/ml; 216-MCC, R&D Systems) to drive macrophage differentiation. For osteoclast differentiation, cells at 70% to 80% confluency were cultured in osteoclast induction medium (high-glucose DMEM with 10% FBS, 1% penicillin/streptomycin, M-CSF [10 ng/ml], and RANKL [100 ng/ml]; 462-TEC, R&D Systems) either with or without DAB, and the culture medium was replaced at 48-h intervals. On day 7, osteoclast formation was evaluated by TRAP staining [56].

BMSCs were isolated using the same protocol as BMDMs. Isolated cells were seeded into 6-well plates and cultured in minimum essential medium α (α-MEM) supplemented with 10% FBS and 1% penicillin/streptomycin. Following a 24-h incubation, the cultures were washed with phosphate-buffered saline (PBS), and the medium was replaced with osteogenic induction medium (α-MEM containing 10% FBS, 1% penicillin/streptomycin, 0.1 μM dexamethasone, ascorbate-2-phosphate [50 μg/ml], and 10 mM β-glycerophosphate). When BMSCs reached 60% to 70% confluence, osteogenic medium (with or without DAB) was added and replaced every 3 d. Osteogenic differentiation was evaluated by ALP staining on day 7 and ARS staining on day 21 [57].

Mouse monocyte/macrophage cell line RAW264.7 (product no. CL-0190) and mouse embryonic osteoblast precursor cell line MC3T3-E1 (product no. CL-0378) were acquired from Wuhan Procell Life Science & Technology Co. Ltd. RAW264.7 cells were cultured in high-glucose DMEM (10% FBS and 1% penicillin/streptomycin); MC3T3-E1 cells were maintained in α-MEM (10% FBS). All cells were incubated in a humidified chamber at 37 °C with 5% CO_2_.

#### Dual-luciferase reporter assay

Cells in logarithmic growth phase were plated in 96-well plates at 1 × 10^4^ cells per well and allowed to reach 70% to 80% confluence. Subsequently, the cultures were cotransfected with either the pRUNX2-TA-Luc or pNF-κB-Luc reporter construct in combination with the *Renilla* luciferase thymidine kinase (pRL-TK) internal control plasmid, using a liposome-based transfection reagent. Following a 48-h incubation period, the cells were lysed, and luciferase activity was quantified with a dual-luciferase reporter assay system, following the protocol provided by the manufacturer. Luc and *Renilla* luciferase (RLuc) activities were detected sequentially [58]. Relative luciferase activity was expressed as the Luc/RLuc ratio, reflecting the transcriptional output of the RUNX2 or NF-κB pathway.

#### CCK-8 assay

The cytotoxicity of DAB against BMSCs was determined using a CCK-8 assay (E-CKK-A362, Elabscience). Following the manufacturer’s protocol, BMSCs were seeded in 96-well plates and exposed to DAB at 0, 1, and 10 μM for 24, 48, and 72 h, with 5 replicates per condition. After each incubation period, 10 μl of CCK-8 reagent was added to each well, incubated at 37 °C for 2 h, and the absorbance at 450 nm was measured on a microplate reader (BioTek, USA).

#### qRT-PCR

Total RNA was harvested from cultured cells by the total RNA isolation reagent method (Thermo Fisher Scientific, USA) per the manufacturer’s guidelines. After purification, cDNA was synthesized using a reverse transcription master mix (Toyobo Co. Ltd., Japan). Amplification for qRT-PCR was then performed on an applied biosystems instrument using SYBR Green PCR master mix (Toyobo Co. Ltd., Japan). Target mRNA expression was quantified relative to a housekeeping gene using the 2^−ΔΔCt^ approach. Five independent biological replicates were included in each analysis.

#### Western blotting

Cellular proteins were extracted with R0010 lysis buffer (Solarbio, Beijing) and their concentrations determined using the P0010 bicinchoninic acid (BCA) protein assay kit (Beyotime). Equal protein amounts were resolved by SDS-PAGE and subsequently electroblotted onto polyvinylidene fluoride membranes (1620177, Bio-Rad). Membranes were blocked for 2 h at room temperature in 5% nonfat milk (P0216, Beyotime) and then probed with primary antibodies overnight at 4 °C. After extensive washing, blots were incubated with appropriate secondary antibodies for 1 h at ambient temperature, and immunoreactive bands were detected with a Tanon imaging system (Shanghai, China).

#### F-actin ring staining

BMDMs were plated into 6-well dishes and exposed to M-CSF (10 ng/ml) plus RANKL (100 ng/ml) for 7 d to promote osteoclast differentiation; parallel cultures were maintained with or without DAB treatment. After differentiation, cells were stained with iFluor 555-Phalloidin (#40737ES, Yeasen, Shanghai, China) to visualize F-actin ring structures. Fluorescent signals were imaged using an Olympus fluorescence microscope, and the proportion of mature osteoclasts was calculated on the basis of the presence of well-defined F-actin rings.

#### Nuclear translocation of NFATc1, TFEB, and RELA

BMSCs and BMDMs were maintained under their respective experimental conditions. For IF staining, cells were fixed with 4% paraformaldehyde for 20 to 30 min, permeabilized in 0.2% Triton X-100 for 2 to 5 min, and blocked for 30 min at room temperature using 5% bovine serum albumin. Subsequently, samples were probed overnight at 4 °C with primary antibodies directed against NFATc1, TFEB, and RELA, followed by a 1-h incubation in the dark with FITC-conjugated goat anti-rabbit IgG (ZF-0316, ZSGB-BIO). Images were captured on an Olympus laser scanning confocal microscope. For Western blot analysis, nuclear and cytoplasmic fractions were prepared using a commercial extraction kit (P0028, Beyotime). Target protein abundance and subcellular distribution were evaluated by Western blotting, with histone-H3 used as the nuclear loading control and GAPDH as the cytoplasmic reference [59,60].

#### TRAP staining

Osteoclast maturation was assessed via TRAP staining. Cultures were fixed with 4% paraformaldehyde and subsequently stained following the manufacturer’s instructions for the commercial TRAP kit (G1050-50T, Servicebio, Wuhan). For quantification, TRAP-positive multinucleated cells containing at least 3 nuclei were identified as mature osteoclasts and enumerated.

#### ARS staining

Following 21 d of osteogenic stimulation, cultures were fixed with 4% paraformaldehyde (Solarbio) and subsequently incubated with alizarin red solution (Solarbio) according to established protocols. The formation of mineralized nodules was visualized and photographed using an Olympus light microscope (Japan) for subsequent analysis.

#### ALP staining

BMSCs and MC3T3-E1 were maintained in osteogenic induction medium for 7 d to evaluate their initial osteogenic potential. Cells were then fixed and incubated with the 5-bromo-4-chloro-3-indolyl phosphate/nitro blue tetrazolium reagent from a commercial ALP assay kit (C3206, Beyotime), which produces a dark purple precipitate at sites of ALP enzymatic activity, enabling direct visualization.

#### ALP activity quantification

ALP activity was quantified using lysates prepared from cultured cells: Cell lysates were prepared using P0013J buffer (Beyotime), and protein content was quantified with the P0012 BCA protein assay kit (Beyotime). ALP activity was subsequently assessed using the P0321 ALP assay kit (Beyotime), which relies on monitoring the hydrolysis of *p*-nitrophenyl phosphate substrate at a wavelength of 405 nm.

#### Bone resorption assay

Osteoclast-mediated bone resorption activity was assessed using flat bovine femur bone slices (#2-0001-100, JoyTech Bio Co., Zhejiang, China). First, BMDMs were seeded onto these slices (placed in 24-well plates) and incubated overnight; the next day, BMDMs were induced in complete medium supplemented with osteoclast differentiation factors and different concentrations of DAB and cultured for 10 d. After induction, the bone slices were fixed in 2.5% glutaraldehyde for 15 min, and adherent cells were removed by intense sonication in 0.25 M ammonium hydroxide. Resorption lacunae on the slice surfaces were then visualized using a SU8100 scanning electron microscope (operated at 3.0 kV); multiple randomly selected fields per slice were imaged to evaluate surface roughness and resorption capacity, and the resorption pit area was measured using ImageJ software according to standard procedures.

#### Coimmunoprecipitation

Co-IP was performed to examine endogenous interactions of RPTOR with p-RPTOR, mTOR, PRKAA, and YWHA, as well as interactions of PRKAA with STK11, CAMKK2, MAP3K7, and RPTOR, in BMSCs under nondenaturing conditions to preserve protein complex integrity. Briefly, BMSCs treated with DAB or vehicle were washed with ice-cold PBS, lysed on ice for 30 min in native lysis buffer supplemented with protease and phosphatase inhibitor cocktails, and centrifuged at 12,000*g* for 15 min at 4 °C. Protein concentrations were determined by BCA assay, and an aliquot of each lysate was retained as the input control. Equal amounts of protein lysates were incubated with anti-RPTOR and anti-PRKAA antibodies or with normal rabbit IgG (isotype control) overnight at 4 °C with gentle rotation, followed by a 4-h incubation with Protein A/G agarose beads to capture immunocomplexes. The beads were then collected, washed 5 times with ice-cold lysis buffer, and boiled in SDS-PAGE loading buffer to elute bound proteins. Eluates were resolved by SDS-PAGE and analyzed by Western blotting to characterize alterations in protein–protein interaction strength upon DAB treatment.

#### NF-κB RELA binding activity assay

BMDMs were plated, grown to the appropriate density, and exposed to varying concentrations of DAB for 1 h prior to stimulation. Nuclear fractions were isolated using a commercial nuclear and cytoplasmic extraction kit (P0028, Beyotime). NF-κB RELA DNA binding activity was then evaluated with a transcription factor ELISA system, following the supplier’s protocol. Briefly, equivalent amounts of nuclear lysate were loaded into microplate wells precoated with oligonucleotides containing an NF-κB consensus binding sequence. Following incubation and subsequent washing, bound RELA was detected by sequential application of a RELA-specific primary antibody and an HRP-linked secondary antibody. Absorbance was read at 450 nm on a microplate reader. All samples were analyzed in triplicate, and blank, negative, and positive controls were run in parallel to verify assay fidelity and reproducibility.

#### ChIP assay

BMDMs were pretreated with DAB and stimulated with TNF-α (100 ng/ml). Cells were fixed using 1% formaldehyde, and the cross-linking reaction was subsequently terminated by the addition of glycine. Chromatin was sheared to an average size of 200 to 500 base pairs and subsequently subjected to immunoprecipitation using an anti-RELA antibody, with normal IgG included as a negative control. Protein–DNA complexes were eluted from the beads, and reversal of cross-linking was subsequently carried out. DNA was then extracted and analyzed by qRT-PCR. The relative enrichment of RELA on the iNOS promoter was calculated. All experiments were repeated independently 5 times.

### In vivo experiment

#### Serum biochemical analysis

At the end of the 60-d in vivo treatment, mice were deeply anesthetized, and blood samples were collected via abdominal aorta puncture. After clotting at room temperature for 30 min, the samples were centrifuged at 3,000*g* for 15 min at 4 °C to separate serum. The serum was then aliquoted and stored at −80 °C until analysis. An automated biochemistry analyzer was used to measure key biochemical parameters reflective of systemic toxicity, including hepatic function markers (alanine transaminase and aspartate transaminase), renal function markers (creatinine and blood urea nitrogen), and metabolic indicators (total cholesterol, triglyceride, and glucose). All assays were conducted in strict accordance with the manufacturers’ protocols.

#### Postmenopausal and natural-aging osteoporosis model

To explore the therapeutic efficacy of DAB, both an OVX-induced postmenopausal model and a natural aging model were established. Twelve-week-old female C57BL/6 mice were randomly allocated into 4 groups (*n* = 6 per group): a sham-operated group, an OVX model group, and 2 OVX subgroups that received daily oral gavage of DAB at doses of 1 and 10 mg/kg. Concurrently, 24-month-old male C57BL/6 mice were randomly assigned to 3 groups (*n* = 6 per group): an equivalent volume of vehicle solvent-treated aging control group and 2 aging subgroups administered daily oral DAB via oral gavage at 1 and 10 mg/kg. For the female cohort, bilateral OVX was performed under aseptic conditions after anesthesia with 3% pentobarbital sodium (35 mg/kg, intraperitoneally). The sham group underwent an identical surgical procedure without ovarian removal. Following a 60-d osteoporosis induction phase, all mice received corresponding drug or vehicle solutions by daily oral gavage for 60 consecutive days. DAB was dissolved in a mixed solvent consisting of DMSO and F-12 medium, while control groups received an equivalent volume of vehicle solvent matched for DMSO and F-12 concentrations. All animals were euthanized upon completion of the 60-d treatment period [61].

#### Micro-CT scanning

Murine femora were fixed in 70% ethanol prior to micro-CT analysis, which was carried out using a QuantumGX2 scanner (PerkinElmer, USA). Imaging parameters were set to an isotropic voxel size of 6 μm, with the x-ray tube operating at 90 kV and 88 mA. For quantitative assessments, a specific volume of interest was selected and positioned 200 sections below the growth plate in the distal metaphysis. Post-3-dimensional (3D) model reconstruction, core morphometric indices including BMD, BV/TV, Tb.N, Tb.Th, and Tb.Sp were quantified.

#### Histology staining (TRAP staining and H&E staining)

Murine femur specimens were collected for histological evaluation. Tissues were fixed in 4% paraformaldehyde for 24 h and then transferred to an ethylenediaminetetraacetic-acid-based decalcification solution (G1105-500ML, Servicebio) until they became palpably pliable, which indicates complete decalcification. After undergoing dehydration and clearing steps, samples were embedded in paraffin and sliced into consecutive 5-μm-thick sections. These sections were processed with standard H&E staining (to assess tissue architecture) and TRAP staining (to specifically visualize osteoclasts) [62]. Stained sections were imaged using a VS200 research-grade optical microscope (Olympus, Japan) to capture digital visuals.

#### IHC staining

IHC staining was performed on 5-μm-thick consecutive paraffin sections of murine femoral tissues (previously decalcified and embedded). The protocol began with deparaffinization, rehydration, and antigen retrieval via a pepsin-containing solution (X1035, Solarbio). Next, endogenous peroxidase activity was quenched, and sections were blocked with 5% bovine serum albumin. Primary antibodies were incubated with the sections overnight at 4 °C. On the subsequent day, immune complexes were detected using the PV-9000 2-step kit (ZSGB-BIO): This involved sequential treatment with a biotin-labeled secondary antibody and streptavidin-HRP, with diaminobenzidine (ZLI-9018, ZSGB-BIO) serving as the chromogenic substrate. Finally, sections were counterstained with hematoxylin to visualize nuclei. Digital images were captured using an Olympus VS200 microscope (Japan), and semiquantitative analysis of DAB signal intensity was conducted via ImageJ software.

#### Calcein double labeling

To assess dynamic bone formation potential, experimental mice were administered 2 intraperitoneal injections of fluorescent labels at distinct time points prior to euthanasia: Calcein (5 mg/kg of body weight) was delivered 10 d before euthanasia, and calcein was given as the second injection 3 d before euthanasia [63]. Following euthanasia, undecalcified bone specimens were embedded in methyl methacrylate resin for subsequent processing. Tissue sections were prepared and visualized under a fluorescence microscope (model VS200, Olympus, Tokyo, Japan) to identify the double-labeled fluorescent bands; these observations were then used to quantify the MAR and BFR/BS.

#### ELISA

Serum levels of P1NP, OCN, CTSK, and ACP5 in mice were quantified using target-specific ELISA kits (Elabscience, Wuhan, China). All procedures were carried out in accordance with the manufacturer’s guidelines. Following completion of the assay, absorbance at 450 nm was measured using a FlexStation 3 multimode microplate reader (Molecular Devices, USA). Absorbance readings were converted to sample concentrations via interpolation from a calibrated reference curve. All subsequent data analysis was conducted in GraphPad Prism 10 software.

### Binding of DAB to PRKAB1 and RELA

#### LiP-MS

Protein lysates were prepared in native buffer with protease inhibitors and quantified by Bradford assay. For limited proteolysis, samples were incubated with Proteinase K (1:100 enzyme-to-substrate ratio, 1 min, 25 °C), followed by heat inactivation. Both LiP and control (trypsin-only) samples were denatured, reduced, alkylated, and sequentially digested with Lys-C and trypsin. Peptides were desalted and analyzed on a Q Exactive HF-X mass spectrometer coupled to an EASY-nLC 1200 system using a 90-min acetonitrile gradient. Full MS scans were acquired at 120,000 resolution (mass/charge ratio [*m*/*z*], 350 to 1,500), and the top 40 precursors underwent higher-energy collisional dissociation fragmentation (27% normalized collision energy) with fragment spectra collected at 15,000 resolution. Protein identification and label-free quantification were performed using Proteome Discoverer with Sequest HT against the UniProtKB Mus musculus database, applying a 1% false discovery rate at both peptide and protein levels [64].

#### DARTS

The DARTS assay was executed using the following procedure. BMDMs or BMSCs were lysed utilizing the M-PER Mammalian Protein Extraction Reagent (#78505, Thermo Fisher Scientific). The obtained lysates were subjected to centrifugation to eliminate insoluble precipitates, and the resulting supernatants were incubated with either DAB or DMSO (serving as the vehicle control) for 1 h at ambient temperature. Next, limited proteolysis was initiated by adding pronase E (#HY-114158, MedChemExpress), with the reaction proceeding for 15 min. To terminate the proteolytic reaction, samples were transferred to ice and supplemented with a deacetylase inhibitor cocktail (P1112, Beyotime) [65].

#### CETSA

CETSA was performed to further validate the direct binding of DAB to target proteins in intact cells, following a standard protocol [66]. For the melting curve assay, BMSCs or BMDMs were treated with 10 μM DAB or vehicle control for 1 h at 37 °C. Cells were harvested, washed with ice-cold PBS, resuspended in PBS supplemented with protease inhibitor cocktail, and divided into 10 aliquots. Each aliquot was heated at a gradient temperature (40 to 75 °C) for 3 min using a PCR thermal cycler, followed by 3 min of cooling at room temperature. Cells were lysed via 3 freeze–thaw cycles (liquid nitrogen/37 °C water bath) and centrifuged at 20,000*g* for 20 min at 4 °C to collect the soluble protein fraction. The level of target protein in the soluble fraction was detected via Western blot. The melting temperature was calculated via nonlinear fitting of the thermal denaturation curve using GraphPad Prism 10.

For the isothermal dose–response assay, cells were treated with gradient concentrations of DAB (0.001 to 100 μM) for 1 h at 37 °C and then heated at a fixed temperature (50 °C for PRKAB1, 60 °C for RELA) for 3 min. The soluble protein fraction was collected, and target protein levels were detected via Western blot. The EC_50_ was calculated via nonlinear fitting using GraphPad Prism 10.

#### MST

Recombinant RELA and PRKAB1 proteins were labeled using the Protein Labeling Kit RED-NHS (NanoTemper Technologies, Beijing, China). Labeled proteins were mixed with a serial dilution of DAB and loaded into Monolith NT.115 capillaries. MST measurements were performed at 40% infrared laser power, and the dissociation constant was calculated using MO.Affinity Analysis software (NanoTemper Technologies).

#### Molecular docking

The 3D structures of target proteins (Q04206 and Q9Y478 from UniProt) were preprocessed using UCSF Chimera to remove water molecules and irrelevant heteroatoms; atomic charges were then calculated with the AMBER14SB force field, and negative logarithm of the acid dissociation constant values were assigned under neutral pH via the H++3 online tool. For the small molecule DAB, 3D structure generation, conformational sampling, and energy minimization were performed with the MMFF94 force field using RDKit, while AM1-BCC partial charges were assigned using UCSF Chimera. Molecular docking was conducted with AutoDock4.2, using a global search strategy and flexible docking mode, with grid box parameters set as follows: for Q04206-DAB, size_x = 56 Å, size_y = 32 Å, size_z = 52 Å, spacing = 0.375 Å, and center coordinates = (−3.847, 2.062, 4.077); for Q9Y478-DAB, size_x = 66.0 Å, size_y = 48.0 Å, size_z = 70.0 Å, spacing = 1 Å, and center coordinates = (−2.836, 10.478, −7.124), and a maximum of 9 conformations were searched using the genetic algorithm. Docking conformations were ranked by scores, and the optimal complexes were analyzed for binding interactions, including hydrogen bonds, hydrophobic interactions, polar interactions, and electrostatic interactions, with PyMOL2.04 used for 3D visualization and Maestro12.8 used for 2D detailed characterization of these interactions [67]*.*

#### CRISPR–Cas9 knockout and siRNA knockdown

##### CRISPR–Cas9

Lentiviral vectors coexpressing Cas9 and single-guide RNAs targeting PRKAB1 or RELA were used to generate knockout cell lines in MC3T3-E1 and RAW264.7 cells. Puromycin-selected clones were validated by Western blotting.

##### siRNA

BMSCs and BMDMs were transfected with specific siRNAs targeting PRKAB1 or RELA or nontargeting control siRNA using Lipo8000 transfection reagent. Knockdown efficiency was assessed 48 h posttransfection.

#### Site-directed mutagenesis and rescue assays

Mutant constructs (PRKAB1-Ala77 and RELA-Arg50) were generated by site-directed mutagenesis PCR and subcloned into the pcDNA3.1(+) vector. Constructs were verified by Sanger sequencing. Knockout or knockdown cells were transfected with wild-type or mutant plasmids using Lipo8000. After 48 h, cells were processed for downstream functional assays and Western blotting.

### Statistics and reproducibility

The sample size (denoted as *n*, referring to the count of biologically independent samples per group) was established by integrating preliminary experimental outcomes, findings from prior studies, and projections of data variability; the specific value for each analysis is detailed in the corresponding figure legend. Throughout data collection and analytical procedures, blinding and random assignment of experimental subjects were adopted to minimize potential bias. All statistical computations and data visualizations were performed using GraphPad Prism 10 (CA, USA). The normality distribution of continuous outcomes was assessed using both the Shapiro–Wilk normality test and quantile–quantile plots, and Levene’s test was implemented to evaluate the homogeneity of variances across groups. Normally distributed data are expressed as means ± SD, whereas nonnormally distributed data are presented as median with interquartile range. For all graphical comparisons between experimental groups, mean values are plotted alongside their corresponding 95% confidence intervals. For comparisons between 2 independent groups that satisfied parametric assumptions, an unpaired 2-tailed Student’s *t* test was applied; when these assumptions were not met, the nonparametric Mann–Whitney *U* test was used instead. One-way or 2-way analysis of variance (ANOVA) was chosen according to the specific experimental design, with Tukey’s honestly significant difference post hoc test applied for pairwise comparisons when ANOVA yielded a significant result. In cases where parametric assumptions could not be satisfied even after appropriate data transformation, the nonparametric Kruskal–Wallis test was utilized, followed by Dunn’s post hoc test with Holm’s correction to account for multiple comparisons. A 2-tailed *P* value less than 0.05 was considered statistically significant. All exact *P* values, the precise statistical tests used, effect size estimates (where calculable), and detailed replication information are provided in the respective figure legends. Every experiment was independently replicated a minimum of 5 times, and all procedures were carried out in compliance with the relevant institutional and national ethical guidelines.

## Ethical Approval

All institutional and national guidelines for the care and use of animals were followed. All procedures about the animal experiments had been approved by the Medical Ethical Committee of Qilu Hospital of Shandong University [approval no. KYLL-2024(ZM)-1243].

## Data Availability

All data associated with this study are available within the paper or the Supplementary Materials. Data are available from the corresponding author upon reasonable request.

## References

[1] Solling AS, Langdahl BL, Cosman F. Recent advances in osteoporosis therapeutics. Annu Rev Med. 2026;77:433–448. 41252575 10.1146/annurev-med-050124-040555

[2] GBD 2019 Fracture Collaborators. Global, regional, and national burden of bone fractures in 204 countries and territories, 1990-2019: A systematic analysis from the Global Burden of Disease Study 2019. Lancet Healthy Longev. 2021;2(9):e580–e592. 34723233 10.1016/S2666-7568(21)00172-0 PMC8547262

[3] Fuggle N, Laslop A, Rizzoli R, Al-Daghri N, Alokail M, Balkowiec-Iskra E, Beaudart C, Bruyère O, Bemden AB, Burlet N, et al. Treatment of osteoporosis and osteoarthritis in the oldest old. Drugs. 2025;85(3):343–360. 39969778 10.1007/s40265-024-02138-w PMC11891106

[4] Zhang L, Guan Q, Wang Z, Feng J, Zou J, Gao B. Consequences of aging on bone. Aging Dis. 2023;15(6):2417–2452. 38029404 10.14336/AD.2023.1115 PMC11567267

[5] Lin Y, Jiang S, Yao Y, Li H, Jin H, Yang G, Ji B, Li Y. Posttranslational modification in bone homeostasis and osteoporosis. MedComm. 2025;6(4): Article e70159. 40170748 10.1002/mco2.70159 PMC11959162

[6] Zhang YW, Wu Y, Liu XF, Chen X, Su JC. Targeting the gut microbiota-related metabolites for osteoporosis: The inextricable connection of gut-bone axis. Ageing Res Rev. 2024;94: Article 102196. 38218463 10.1016/j.arr.2024.102196

[7] Liu RX, Gu RH, Li ZP, Hao ZQ, Hu QX, Li ZY, Wang XG, Tang W, Wang XH, Zeng YK, et al. Trim21 depletion alleviates bone loss in osteoporosis via activation of YAP1/β-catenin signaling. Bone Res. 2023;11(1):56. 37884520 10.1038/s41413-023-00296-3 PMC10603047

[8] Sun X, Wu T, Chen S, Zhao Z, Jia R, Ma J, Yin L, Pan X, Ping Y, Mao Y, et al. OTUD1 inhibits osteoclast differentiation and osteoclastic bone loss through deubiquitinating and stabilizing PRDX1. Theranostics. 2025;15(14):6719–6736. 40585986 10.7150/thno.111360 PMC12203675

[9] Tan L, Tu Y, Miao Z, Zhao Y, Liang Y, Zhong J, Zhong R, Xu N, Chen X, He C. Glycyrol alleviates osteoporosis through dual modulation on osteoclastogenesis and osteogenesis by targeting Syk signaling pathway. Phytomedicine. 2025;138: Article 156429. 39939034 10.1016/j.phymed.2025.156429

[10] Handel MN, Cardoso I, Von Bülow C, Rohde JF, Ussing A, Nielsen SM, Christensen R, Body JJ, Brandi ML, Diez-Perez A, et al. Fracture risk reduction and safety by osteoporosis treatment compared with placebo or active comparator in postmenopausal women: Systematic review, network meta-analysis, and meta-regression analysis of randomised clinical trials. BMJ. 2023;381: Article e068033. 37130601 10.1136/bmj-2021-068033 PMC10152340

[11] Wang H, Luo Y, Wang H, Li F, Yu F, Ye L. Mechanistic advances in osteoporosis and anti-osteoporosis therapies. MedComm. 2023;4: Article e244. 37188325 10.1002/mco2.244 PMC10175743

[12] Beth-Tasdogan NH, Mayer B, Hussein H, Zolk O, Peter JU. Interventions for managing medication-related osteonecrosis of the jaw. Cochrane Database Syst Rev. 2022;7(7):CD012432. 35866376 10.1002/14651858.CD012432.pub3 PMC9309005

[13] McClung MR. Considerations regarding the use of romosozumab in a patient at high risk for cardiovascular disease. J Bone Miner Res. 2026;41(4):382–386. 41189403 10.1093/jbmr/zjaf164

[14] Sopina L, Hitz MF, Thygesen LC, Langdahl B, Ladefoged BT, Kruse M. Healthcare and productivity cost of osteoporosis: A Danish register-based quasi-experimental study. Osteoporos Int. 2025;36(5):865–874. 40111480 10.1007/s00198-025-07453-w PMC12089169

[15] GBD 2021 Low Bone Mineral Density Collaborators. The global, regional, and national burden attributable to low bone mineral density, 1990-2020: An analysis of a modifiable risk factor from the Global Burden of Disease Study 2021. Lancet Rheumatol. 2025;7(12):e873–e894. 40972625 10.1016/S2665-9913(25)00105-5 PMC12623303

[16] Zhang YW, Li RY, Wu Y, Wang P, Zhou QR, Su JC. Gut microbiota and bone aging: Focusing on the gut-X axis modes. J Orthop Translat. 2026;57: Article 101064. 41777702 10.1016/j.jot.2026.101064 PMC12950467

[17] Zhang YW, Cao MM, Li YJ, Dai GC, Lu PP, Zhang M, Bai LY, Chen XX, Zhang C, Shi L, et al. The regulative effect and repercussion of probiotics and prebiotics on osteoporosis: Involvement of brain-gut-bone axis. Crit Rev Food Sci Nutr. 2023;63(25):7510–7528. 35234534 10.1080/10408398.2022.2047005

[18] Brent MB. Pharmaceutical treatment of bone loss: From animal models and drug development to future treatment strategies. Pharmacol Ther. 2023;244: Article 108383. 36933702 10.1016/j.pharmthera.2023.108383

[19] Wen S, Liao X, Chang R, Wang S. Structures and biological activities of anti-osteoporotic drugs: An overview of promising small-molecule therapeutics for the treatment of osteoporosis. Bioorg Chem. 2025;162: Article 108568. 40381465 10.1016/j.bioorg.2025.108568

[20] Douketis JD, Spyropoulos AC. Perioperative management of patients taking direct oral anticoagulants: A review. JAMA. 2024;332(10):825–834. 39133476 10.1001/jama.2024.12708

[21] He N, Dell’Aniello S, Zhai S, Suissa S, Renoux C. Risk of fracture in patients with non-valvular atrial fibrillation initiating direct oral anticoagulants vs. vitamin K antagonists. Eur Heart J Cardiovasc Pharmacother. 2021;7(5):389–397. 32722764 10.1093/ehjcvp/pvaa094 PMC8453296

[22] Yang N, Zhao Y, Bai Z, Chen H, Ning H, Zou M, Cheng G. The association of non-vitamin K antagonist oral anticoagulants vs. warfarin and the risk of fractures for patients with atrial fibrillation: A systematic review and meta-analysis. Acta Cardiol. 2023;78(3):298–310. 36063197 10.1080/00015385.2022.2030555

[23] Chen Z, Xie H, Yuan J, Lan Y, Xie Z. Kruppel-like factor 6 promotes odontoblastic differentiation through regulating the expression of dentine sialophosphoprotein and dentine matrix protein 1 genes. Int Endod J. 2021;54(4):572–584. 33200415 10.1111/iej.13447

[24] Fu W, Chen M, Wang K, Chen Y, Cui Y, Xie Y, Lei ZN, Hu W, Sun G, Huang G, et al. Tau is a receptor with low affinity for glucocorticoids and is required for glucocorticoid-induced bone loss. Cell Res. 2025;35(1):23–44. 39743632 10.1038/s41422-024-01016-0 PMC11701132

[25] Wang J, Zhang Y, Cao J, Wang Y, Anwar N, Zhang Z, Zhang D, Ma Y, Xiao Y, Xiao L, et al. The role of autophagy in bone metabolism and clinical significance. Autophagy. 2023;19(9):2409–2427. 36858962 10.1080/15548627.2023.2186112 PMC10392742

[26] Tan A, Prasad R, Lee C, Jho EH. Past, present, and future perspectives of transcription factor EB (TFEB): Mechanisms of regulation and association with disease. Cell Death Differ. 2022;29(8):1433–1449. 35739255 10.1038/s41418-022-01028-6 PMC9345944

[27] Giamogante F, Barazzuol L, Maiorca F, Poggio E, Esposito A, Masato A, Napolitano G, Vagnoni A, Calì T, Brini M. A SPLICS reporter reveals α-synuclein regulation of lysosome-mitochondria contacts which affects TFEB nuclear translocation. Nat Commun. 2024;15(1):1516. 38374070 10.1038/s41467-024-46007-2 PMC10876553

[28] Cui Z, Napolitano G, De Araujo ME, Esposito A, Monfregola J, Huber LA, Ballabio A, Hurley JH. Structure of the lysosomal mTORC1-TFEB-Rag-Ragulator megacomplex. Nature. 2023;614(7948):572–579. 36697823 10.1038/s41586-022-05652-7 PMC9931586

[29] Robichaud S, Fairman G, Vijithakumar V, Mak E, Cook DP, Pelletier AR, Huard S, Vanderhyden BC, Figeys D, Lavallée-Adam M, et al. Identification of novel lipid droplet factors that regulate lipophagy and cholesterol efflux in macrophage foam cells. Autophagy. 2021;17(11):3671–3689. 33590792 10.1080/15548627.2021.1886839 PMC8632324

[30] Penugurti V, Manne RK, Bai L, Kant R, Lin HK. AMPK: The energy sensor at the crossroads of aging and cancer. Semin Cancer Biol. 2024;106-107:15–27. 39197808 10.1016/j.semcancer.2024.08.002 PMC11625618

[31] Reid IR, Billington EO. Drug therapy for osteoporosis in older adults. Lancet. 2022;399(10329):1080–1092. 35279261 10.1016/S0140-6736(21)02646-5

[32] Xie Q, Du X, Liang J, Shen Y, Ling Y, Huang Z, Ke Z, Li T, Song B, Wu T, et al. FABP4 inhibition suppresses bone resorption and protects against postmenopausal osteoporosis in ovariectomized mice. Nat Commun. 2025;16(1):4437. 40360512 10.1038/s41467-025-59719-w PMC12075751

[33] Wu M, Wu S, Chen W, Li YP. The roles and regulatory mechanisms of TGF-β and BMP signaling in bone and cartilage development, homeostasis and disease. Cell Res. 2024;34(2):101–123. 38267638 10.1038/s41422-023-00918-9 PMC10837209

[34] Shen Y, Huang X, Wu J, Lin X, Zhou X, Zhu Z, Pan X, Xu J, Qiao J, Zhang T, et al. The global burden of osteoporosis, low bone mass, and its related fracture in 204 countries and territories, 1990-2019. Front Endocrinol. 2022;13: Article 882241. 10.3389/fendo.2022.882241 PMC9165055 35669691

[35] Chen YJ, Jia LH, Han TH, Zhao ZH, Yang J, Xiao JP, Yang HJ, Yang K. Osteoporosis treatment: Current drugs and future developments. Front Pharmacol. 2024;15:1456796. 39188952 10.3389/fphar.2024.1456796 PMC11345277

[36] Billington E, Aghajafari F, Skulsky E, Kline GA. Bisphosphonates. BMJ. 2024;386: Article e076898. 39168493 10.1136/bmj-2023-076898

[37] Wen MT, Li JC, Lu BW, Shao HR, Ling PX, Liu F, Li G, Luo D. Indications and adverse events of teriparatide: Based on FDA adverse event reporting system (FAERS). Front Pharmacol. 2024;15:1391356. 39170708 10.3389/fphar.2024.1391356 PMC11335658

[38] Xiao Y, Chen Y, Huang Y, Xiao Y. Atypical femur fracture associated with common anti-osteoporosis drugs in FDA adverse event reporting system. Sci Rep. 2023;13(1):10892. 37407650 10.1038/s41598-023-37944-x PMC10322871

[39] Lau WC, Chan EW, Cheung CL, Sing CW, Man KK, Lip GY, Siu CW, Lam JK, Lee AC, Wong IC. Association between dabigatran vs warfarin and risk of osteoporotic fractures among patients with nonvalvular atrial fibrillation. JAMA. 2017;317(11):1151–1158. 28324091 10.1001/jama.2017.1363

[40] Huang C, Chen X, Wu D, Chen J, Wang J, Li S, Hu J, Yan Z, Zhu Y, Zhang Y. Autophagic damage in senescent bone marrow mesenchymal stromal cells: Impact on Piezo1 expression during osteoporosis progression. Int J Biol Macromol. 2025;330: Article 147928. 41015372 10.1016/j.ijbiomac.2025.147928

[41] Xue H, Feng Z, Yuan P, Qiao L, Lou Q, Zhao X, Ma Q, Wang S, Shen Y, Ye H, et al. Restrained Mitf-associated autophagy by mulberroside A ameliorates osteoclastogenesis and counteracts OVX-induced osteoporosis in mice. Cell Death Discov. 2024;10(1):80. 38360705 10.1038/s41420-024-01847-1 PMC10869803

[42] Bai L, Liu Y, Zhang X, Chen P, Hang R, Xiao Y, Wang J, Liu C. Osteoporosis remission via an anti-inflammaging effect by icariin activated autophagy. Biomaterials. 2023;297: Article 122125. 37058900 10.1016/j.biomaterials.2023.122125

[43] Zhang S, Xie Y, Yan F, Zhang Y, Yang Z, Chen Z, Zhao Y, Huang Z, Cai L, Deng Z. Negative pressure wound therapy improves bone regeneration by promoting osteogenic differentiation via the AMPK-ULK1-autophagy axis. Autophagy. 2022;18(9):2229–2245. 34964701 10.1080/15548627.2021.2016231 PMC9466622

[44] Ma T, Tian X, Zhang B, Li M, Wang Y, Yang C, Wu J, Wei X, Qu Q, Yu Y, et al. Low-dose metformin targets the lysosomal AMPK pathway through PEN2. Nature. 2022;603(7899):159–165. 35197629 10.1038/s41586-022-04431-8 PMC8891018

[45] Visnjic D, Lalic H, Dembitz V, Tomic B, Smoljo T. AICAr, a widely used AMPK activator with important AMPK-independent effects: A systematic review. Cells. 2021;10(5):1095. 34064363 10.3390/cells10051095 PMC8147799

[46] Paquette M, El-Houjeiri L, Zirden L, Puustinen P, Blanchette P, Jeong H, Dejgaard K, Siegel PM, Pause A. AMPK-dependent phosphorylation is required for transcriptional activation of TFEB and TFE3. Autophagy. 2021;17(12):3957–3975. 33734022 10.1080/15548627.2021.1898748 PMC8726606

[47] Kazyken D, Dame SG, Wang C, Wadley M, Fingar DC. Unexpected roles for AMPK in the suppression of autophagy and the reactivation of MTORC1 signaling during prolonged amino acid deprivation. Autophagy. 2024;20(9):2017–2040. 38744665 10.1080/15548627.2024.2355074 PMC11346535

[48] Goul C, Peruzzo R, Zoncu R. The molecular basis of nutrient sensing and signalling by mTORC1 in metabolism regulation and disease. Nat Rev Mol Cell Biol. 2023;24(12):857–875. 37612414 10.1038/s41580-023-00641-8

[49] Jin Y, Wu O, Chen Q, Chen L, Zhang Z, Tian H, Zhou H, Zhang K, Gao J, Wang X, et al. Hypoxia-preconditioned BMSC-derived exosomes induce mitophagy via the BNIP3-ANAX2 axis to alleviate intervertebral disc degeneration. Adv Sci. 2024;11(34): Article e2404275. 10.1002/advs.202404275 PMC11425632 38973294

[50] Tao H, Li W, Zhang W, Yang C, Zhang C, Liang X, Yin J, Bai J, Ge G, Zhang H, et al. Urolithin A suppresses RANKL-induced osteoclastogenesis and postmenopausal osteoporosis by, suppresses inflammation and downstream NF-κB activated pyroptosis pathways. Pharmacol Res. 2021;174: Article 105967. 34740817 10.1016/j.phrs.2021.105967

[51] Zhang Y, He X, Wang K, Xue Y, Hu S, Jin Y, Zhu G, Shi Q, Rui Y. Irisin alleviates obesity-induced bone loss by inhibiting interleukin 6 expression via TLR4/MyD88/NF-κB axis in adipocytes. J Adv Res. 2025;69:343–359. 38626873 10.1016/j.jare.2024.04.013 PMC11954833

[52] Guo Q, Jin Y, Chen X, Ye X, Shen X, Lin M, Zeng C, Zhou T, Zhang J. NF-κB in biology and targeted therapy: New insights and translational implications. Signal Transduct Target Ther. 2024;9(1):53. 38433280 10.1038/s41392-024-01757-9 PMC10910037

[53] Ramsey KM, Dembinski HE, Chen W, Ricci CG, Komives EA. DNA and IκBα both induce long-range conformational changes in NFκB. J Mol Biol. 2017;429(7):999–1008. 28249778 10.1016/j.jmb.2017.02.017 PMC5389416

[54] Eikelboom JW, Wallentin L, Connolly SJ, Ezekowitz M, Healey JS, Oldgren J, Yang S, Alings M, Kaatz S, Hohnloser SH, et al. Risk of bleeding with 2 doses of dabigatran compared with warfarin in older and younger patients with atrial fibrillation: An analysis of the randomized evaluation of long-term anticoagulant therapy (RE-LY) trial. Circulation. 2011;123(21):2363–2372. 21576658 10.1161/CIRCULATIONAHA.110.004747

[55] Liu F, Yuan Y, Bai L, Yuan L, Li L, Liu J, Chen Y, Lu Y, Cheng J, Zhang J. LRRc17 controls BMSC senescence via mitophagy and inhibits the therapeutic effect of BMSCs on ovariectomy-induced bone loss. Redox Biol. 2021;43: Article 101963. 33865167 10.1016/j.redox.2021.101963 PMC8066428

[56] Liu K, Wang Z, Liu J, Zhao W, Qiao F, He Q, Shi J, Meng Q, Wei J, Cheng L. Atsttrin regulates osteoblastogenesis and osteoclastogenesis through the TNFR pathway. Commun Biol. 2023;6(1):1251. 38081906 10.1038/s42003-023-05635-y PMC10713527

[57] Zhang F, Wang T, Wei L, Xie Z, Wang L, Luo H, Li F, Kang Q, Dong W, Zhang J, et al. B-lymphoid tyrosine kinase crosslinks redox and apoptosis signaling networks to promote the survival of transplanted bone marrow mesenchymal stem cells. Research. 2025;8:0660. 40235595 10.34133/research.0660 PMC11999575

[58] Tao M, Wang L, Chen C, Tang M, Wang Y, Zhang J, Zhao X, Feng Q, Chen J, Yan W, et al. Developmentally endothelial locus-1 facilitates intestinal inflammation resolution by suppressing the Cmpk2-cGAS-STING pathway and promoting reparatory macrophage transition. J Adv Res. 2026;80:593–608. 40288675 10.1016/j.jare.2025.04.030 PMC12869262

[59] Xiu C, Luo H, Huang W, Fan S, Yuan C, Chen J, Xu C, Yao C, Hong D, Zhang L. Lobetyolin suppressed osteoclastogenesis and alleviated bone loss in ovariectomy-induced osteoporosis via hindering p50/p65 nuclear translocation and downstream NFATc1/c-Fos expression. Drug Des Devel Ther. 2025;19:4689–4715. 10.2147/DDDT.S515930 PMC12145116 40486125

[60] Deng W, Ding Z, Wang Y, Zou B, Zheng J, Tan Y, Yang Q, Ke M, Chen Y, Wang S, et al. Dendrobine attenuates osteoclast differentiation through modulating ROS/NFATc1/ MMP9 pathway and prevents inflammatory bone destruction. Phytomedicine. 2022;96: Article 153838. 34801352 10.1016/j.phymed.2021.153838

[61] Ruan B, Dong J, Wei F, Huang Z, Yang B, Zhang L, Li C, Dong H, Cao W, Wang H, et al. DNMT aberration-incurred GPX4 suppression prompts osteoblast ferroptosis and osteoporosis. Bone Res. 2024;12(1):68. 39617773 10.1038/s41413-024-00365-1 PMC11609303

[62] Yuan P, Feng Z, Yang H, Xue H, Xie H, Dai Z, Wang H, Liu Y, Pan B, Song H, et al. Visomitin attenuates pathological bone loss by reprogramming osteoclast metabolism via the STAT3/LDHB axis. Research. 2025;8:0784. 40698330 10.34133/research.0784 PMC12280330

[63] Zhang L, Qi B, Li Y, Liang X, Zhang Z, Yang T, Jia S, Gao X, Chen S, Jiao G, et al. GLUL mediates FOXO3 O-GlcNAcylation to regulate the osteogenic differentiation of BMSCs and senile osteoporosis. Cell Death Differ. 2025;32(12):2399–2411. 40646162 10.1038/s41418-025-01543-2 PMC12669651

[64] Chen S, Liu X, Peng C, Tan C, Sun H, Liu H, Zhang Y, Wu P, Cui C, Liu C, et al. The phytochemical hyperforin triggers thermogenesis in adipose tissue via a Dlat-AMPK signaling axis to curb obesity. Cell Metab. 2021;33(3):565–580 e567. 33657393 10.1016/j.cmet.2021.02.007

[65] Liu K, Zhang X, Ning B, Liu R, Wang C, Cheng L, Zheng W, Wei J. Fexofenadine protects against osteoarthritis by targeting Smad2 and STAT1 to enhance anabolism and binding cPLA2 to inhibit catabolism. Cell Death Discov. 2025;11(1):473. 41120290 10.1038/s41420-025-02754-9 PMC12540828

[66] Wang KD, Ding X, Jiang N, Zeng C, Wu J, Cai XY, Hettinghouse A, Khleborodova A, Lei ZN, Chen ZS, et al. Digoxin targets low density lipoprotein receptor-related protein 4 and protects against osteoarthritis. Ann Rheum Dis. 2022;81(4):544–555. 34853001 10.1136/annrheumdis-2021-221380 PMC9082564

[67] Liu R, Chen Y, Fu W, Wang S, Cui Y, Zhao X, Lei ZN, Hettinghouse A, Liu J, Wang C, et al. Fexofenadine inhibits TNF signaling through targeting to cytosolic phospholipase A2 and is therapeutic against inflammatory arthritis. Ann Rheum Dis. 2019;78(11):1524–1535. 31302596 10.1136/annrheumdis-2019-215543 PMC8157820

